# Magnetic resonance imaging of noradrenergic neurons

**DOI:** 10.1007/s00429-019-01858-0

**Published:** 2019-03-22

**Authors:** Takashi Watanabe, Zhengguo Tan, Xiaoqing Wang, Ana Martinez-Hernandez, Jens Frahm

**Affiliations:** 1 Biomedizinische NMR, Max-Planck-Institut für biophysikalische Chemie, 37077 Göttingen, Germany; 2 Abteilung Gene und Verhalten, Max-Planck-Institut für biophysikalische Chemie, 37077 Göttingen, Germany

**Keywords:** Alzheimer’s disease, Dorsal motor vagus nucleus, Locus coeruleus, Magnetization transfer, Neuromelanin, Nucleus tractus solitarius

## Abstract

Noradrenaline is a neurotransmitter involved in general arousal, selective attention, memory, inflammation, and neurodegeneration. The purpose of this work was to delineate noradrenergic neurons in vivo by *T*_1_-weighted MRI with magnetization transfer (MT). In the brainstem of human and mice, MRI identified the locus coeruleus, dorsal motor vagus nucleus, and nucleus tractus solitarius. Given (1) the long *T*_1_ and low magnetization transfer ratio for the noradrenergic cell groups compared to other gray matter, (2) significant correlation between MT MRI signal intensity and proton density, and (3) no correlation between magnetization transfer ratio (or *R*_1_) and iron, copper, or manganese in human brain, the high MRI signal of the noradrenergic neurons must be attributed to abundant water protons interacting with any *T*_1_-shortening paramagnetic ions in active cells rather than to specific *T*_1_-shortening molecules. The absence of a high MRI signal from the locus coeruleus of Ear2(−/−) mice lacking noradrenergic neurons confirms that cell bodies of noradrenergic neurons are the source of the bright MRI appearance. The observation of this high signal in DBH(−/−) mice, in 3-week-old mice, and in mice under hyperoxia/hypercapnia/hypoxia together with the general absence of neuromelanin (NM) in noradrenergic neurons of young rodents further excludes that it is due to NM, dopamine β-hydroxylase, their binding to paramagnetic ions, blood inflow, or hemoglobin. Instead, these findings indicate a high density of water protons whose *T*_1_ is shortened by paramagnetic ions as the relevant source of the high MRI signal. In the brain of APP/PS1/Ear2(−/−) mice, a transgenic model of Alzheimer’s disease, MRI detected noradrenergic neuron loss in the locus coeruleus. Proton magnetic resonance spectroscopy revealed that a 60–75% reduction of noradrenaline is responsible for a reduction of *N*-acetylaspartate and glutamate in the hippocampus as well as for a shortening of the water proton *T*_2_ in the frontal cortex. These results suggest that a concurrent shortage of noradrenaline in Alzheimer’s disease accelerates pathologic processes such as inflammation and neuron loss.

**Electronic supplementary material:**

The online version of this article (10.1007/s00429-019-01858-0) contains supplementary material, which is available to authorized users.

## Introduction

Noradrenaline (NA), also called norepinephrine, is a catecholamine that acts as hormone and neurotransmitter. It is involved in general arousal, selective attention, memory, and stress reactivity as well as in inflammation and neurodegeneration, e.g., in Alzheimer’s disease (Heneka et al. 2015; Kummer et al. 2014). Noradrenergic neurons (NA neurons), which produce the neuromodulator and release it from axonal terminals that spread widely over the brain, are assembled in the brainstem as A1–A7 cell groups (Dahlström and Fuxe 1964). The major groups are A2, i.e., the dorsal motor vagus nucleus and the nucleus tractus solitarius in the medulla oblongata, and A6, i.e., the locus coeruleus (LC) in the pons (Moore and Bloom 1978). Cell bodies of NA neurons are rich in copper (Cu^2+^) ions, because Cu^2+^ is required for the electron transfer in an enzymatic reaction catalyzed by dopamine β-hydroxylase that converts dopamine to NA (Kaufman 1974). Accordingly, the copper concentration in LC is 6- to 10-fold higher than in other gray matter or 14- to 20-fold higher than in white matter (WM) (data from human, Prohaska 1987). Thus, NA neurons can be assumed to contain abundant water protons whose *T*_1_ is shortened by paramagnetic ions.

The purpose of this work was to (a) delineate NA neurons in human and mice in vivo by combining *T*_1_-weighted MRI with magnetization transfer (MT), which does not affect the MRI signal increase induced by paramagnetic ions but suppresses signals from water molecules in contact with diamagnetic macromolecules (Henkelman et al. 2001; Watanabe et al. 2012), (b) compare MRI of wild-type and transgenic mice, i.e., Ear2(−/−) and DBH(−/−), to verify the source of the image contrast, (c) apply MRI to characterize APP/PS1/Ear2(−/−) mice, a transgenic model of Alzheimer’s disease, and (d) perform proton magnetic resonance spectroscopy (MRS) to further characterize the transgenic model.

## Materials and methods

### Human brain MRI

All procedures involving human participants (adult male, *n* = 12) in this study were approved by the ethics committee of the Georg-August-Universität Göttingen and performed in accordance with the ethical standards of the institutional and national research committee and with the 1964 Helsinki declaration and its later amendments. All participants gave written informed consent before each examination.

At 3T (Magnetom Prisma, Siemens Healthcare, Erlangen, Germany) transversal MRI (2D FLASH, TR/TE = 863/4.4 ms, pixel bandwidth = 140 Hz, (0.66 mm)^2^ resolution, 2.5 mm slice thickness, 21 slices, total acquisition = 4 min 35 s) was performed with the use of a 64-channel head coil. An on-resonance flip angle *α* of 70° was used for *T*_1_-weighted MRI, while *α* of 15° was used for proton-density-weighted MRI. For off-resonance irradiation, MT (10 ms Gaussian pulse, frequency offset = 1200 Hz, flip angle = 208.5°, amplitude = 27.9 V) and fat saturation were used as provided by the manufacturer. *T*_1_ mapping (Wang et al. 2015, 2018) as well as *T*_2_ and *M*_0_ mapping (model-based accelerated *T*_2_ mapping, Siemens) was performed at the same spatial resolution. Regions-of-interest is selected in the frontal subcortical WM, prefrontal cortex, caudate nucleus, putamen, thalamus, globus pallidus, subthalamic nucleus, red nucleus, and substantia nigra. Regional values are compared to each other and to the mean regional $R_{2}^{*}$ values (Deistung et al. 2013; Tan 2016; Wansapura et al. 1999; Yao et al. 2009) as well as to the mean regional content of water (Gelman et al. 2001; Wood 1982), iron (Hallgren and Sourander 1958), copper (Warren et al. 1960), and manganese (Duflou et al. 1989).

For magnetization transfer gradient-echo MRI of LC and A2, the data were accumulated twice (TR/TE = 863/4.4 ms, total acquisition = 9 min 10 s). The slices are positioned perpendicular to the anterior wall of the fourth ventricle. The LC is observable in the 5th–7th slices from the top, while A2 is observable in the 16th–18th slices. For comparison, interleaved multi-slice *T*_1_-weighted fat-suppressed 2D fast spin-echo MRI at the same spatial resolution was performed with TR/TE = 597/7.3 ms, echo train length = 3, flip angle = 150°, pixel bandwidth = 400 Hz, number of slices = 3 or 21, total acquisition = 6 min 22 s.

### Calculation of signal intensity in gradient-echo MRI of the brain

In MRI of the brain in vivo, the signal originates exclusively from water protons. With negligible signal contributions from *T*_2_ coherence, the observable signal in the steady state of a spoiled gradient-echo sequence yields:

$${S_0}={M_0}\frac{{1 - {e^{ - {\text{TR}} \times {R_1}}}}}{{1 - \cos \alpha {e^{ - {\text{TR}} \times {R_1}}}}}{e^{ - {\text{TE}} \times R_{2}^{*}}},$$

with flip angle *α*, repetition time TR, echo time TE, spin–lattice relaxation rate *R*_1_, effective spin–spin relaxation rate $R_{2}^{*}$, and initial magnetization *M*_0_. The observable signal in magnetization transfer MRI yields: $S={S_{0~}}(1 - {\text{MTR}})$ with *S*_0_ the observable signal in gradient-echo MRI without MT and MTR the magnetization transfer ratio.

### Animals

Mice were housed in groups under standard conditions at a temperature of 22 °C and a 12 h light/dark cycle with ad libitum access to standard food and water. All experiments were performed in accordance with German animal protection laws after approval by the responsible governmental authority. A total of 73 mice were used. Seven Ear2(−/−) mice (three male and four female, 4 weeks old, 12–18 g), 7 C57BL/6N wild-type mice (12–18 g, age-, strain-, as well as gender matched), 5 dopamine β-hydroxylase (+/−), and 5 dopamine β-hydroxylase (−/−) mice were used for the validation of the contrast source. Thirteen aged (≥ 12 months old) (4 male, 9 female) C57BL/6N wild-type mice, 17 aged APP/PS1/Ear2(−/−) (4 male, 13 female) mice, 12 aged APP/PS1 mice (4 male, 8 female), and 4 aged female Ear2(−/−) mice were used for characterizing the animal model of Alzheimer’s disease. Ear2(−/−), APP/PS1, APP/PS1/Ear2(−/−), DBH(+/−), and DBH(−/−) mice were generated as described previously (Hammerschmidt et al. 2013; Jankowsky et al. 2001; Kummer et al. 2014; Warnecke et al. 2005). In addition, one female (11 weeks old, 21 g) and two male (3 weeks old, 9 g and 13 g) C57BL/6N wild-type mice were used for a study at 9.4 T.

In Ear2(−/−) mice, a nuclear hormone receptor Ear2 is lacking, which leads to ~ 70% reduction of LC neurons (Warnecke et al. 2005). In DBH(−/−) mice, the enzyme dopamine β-hydroxylase is lacking in contrast to DBH(+/−) or DBH(+/+) mice (Hammerschmidt et al. 2013; Kummer et al. 2014). APP/PS1 mice, where amyloid precursor protein and presenilin-1 are expressed, serve as a model for the human Alzheimer’s disease (Kummer et al. 2014). In APP/PS1/Ear2(−/−) mice, amyloid precursor protein and presenilin-1 are expressed but the nuclear hormone receptor Ear2 is lacking (Kummer et al. 2014). Consequently, APP/PS1/Ear2(−/−) mice serve as a model for LC degeneration and subsequent NA deficiency in Alzheimer’s disease.

### Anesthesia

After induction of anesthesia with 5% isoflurane, animals were intubated with a purpose-built polyethylene endotracheal tube (0.58 mm inner diameter, 0.96 mm outer diameter) and artificially ventilated using an animal respirator (TSE, Bad Homberg, Germany) with a respiratory rate of 25 breaths per minute and an estimated tidal volume of 0.35 ml as previously described (Schulz et al. 2002; Watanabe et al. 2004, 2016a). The animals were then placed in a prone position on a purpose-built palate holder equipped with an adjustable nose cone. The Göttingen animal bed (Tammer et al. 2007) secured a reproducible and reliable fixation of the mouse head and receiver coil in the magnet isocenter. Respiratory movement of the abdomen as well as rectal temperature was monitored by a unit supplied by the manufacturer (Bruker Biospin MRI GmbH, Ettlingen, Germany).

In addition, to examine whether a change in blood supply influences the MRI signal intensity, the animals were anesthetized by intraperitoneal injection of ketamine (200 mg/kg body weight) and xylazine (16 mg/kg body weight). Hyperoxia was induced by changing the inspiratory gas from ~ 90% air and ~ 10% O_2_ to ~ 20% air and ~ 80% O_2_, while hypoxic hypercapnia was induced by ~ 60% air, ~ 10% O_2_, and ~ 30% CO_2_ as described previously (Watanabe et al. 2016a).

### Mouse brain MRI at 2.35 T

At 2.35 T, MRI measurements were carried out using a 400-mm bore magnet (Magnex Scientific, Abingdon, UK) equipped with 200 mT m^−1^ gradients (Bruker Biospin MRI GmbH, Ettlingen, Germany). RF (radiofrequency) excitation and signal reception were accomplished with the use of a Helmholtz coil (inner diameter 100 mm) and an elliptical surface coil (inner diameter 20 mm × 14 mm), respectively. An off-resonance Gaussian RF pulse with a duration of 12 ms, a frequency offset of 2200–5000 Hz, and a mean amplitude of 50–200 Hz (flip angle 261°–1045°) was incorporated into a gradient-echo MRI sequence (RF-spoiled 3D FLASH, TR/TE = 30/7.6 ms, flip angle = 10°–30°, field-of-view = 30 × 18.75 × 22.5 mm^3^, matrix = 256 × 160 × 192 interpolated to 512 × 512 × 512, 8 averages, total acquisition = 123 min) at 117 µm isotropic resolution. The magnetization transfer (MT) ratio (MTR) was obtained from acquisitions with and without off-resonance irradiation.

For evaluation of signal intensities, anatomically defined cross-sections were obtained from the original 3D MRI data sets by multiplanar reconstructions using software supplied by the manufacturer (Paravision 5.0, Bruker Biospin MRI GmbH, Ettlingen, Germany). The plane of the anterior commissure-posterior commissure served as a reference for the selection of standardized sections to facilitate comparisons with minimized intra- and inter-individual variability. For LC or A2, a rectangular region-of-interest of six pixels was taken in the center of the delineated structures. For the brainstem, a circular region-of-interest of 1004 pixels was taken in the brainstem between LC and A2. The SNR was defined as the mean MRI signal intensity divided by the standard deviation of the noise. The analysis followed a strategy previously developed for intra-individual comparisons of MR images obtained after manganese administration (Watanabe et al. 2004).

### Mouse brain MRI at 9.4 T

High-field MRI measurements were carried out at 9.4 T (Bruker Biospin MRI GmbH, Ettlingen, Germany). RF excitation was accomplished with the use of a birdcage resonator (inner diameter 70 mm), while signals were received by a four-channel phased-array surface coil. An off-resonance Gaussian RF pulse with a frequency offset of − 1.4 kHz, a duration of 12 ms, and a flip angle of 240° was incorporated into a fat-suppressed gradient-echo MRI sequence (3D FLASH) with: TR/TE = 33/7.5 ms, flip angle 30°, spectral bandwidth 21 kHz, field-of-view 20.5 × 15.4 × 20.5 mm^3^, matrix 256 × 192 × 256, at an isotropic resolution of 80 µm, 2 averages, total acquisition 54 min. In addition, fat-suppressed low-resolution MRI (spectral bandwidth 10 kHz, field-of-view (15 mm)^3^, matrix 128^3^, isotropic resolution of 117 µm, 2 averages, total acquisition 9 min) was performed with MT (flip angle of 120°) to examine whether a change in blood supply influences the signal intensity.

### Magnetic resonance spectroscopy

At 9.4 T, localized proton MRS (STEAM, TR/TE/TM = 6000/10/10 ms) was performed with the use of the birdcage resonator and a saddle-shaped quadrature surface coil (Bruker Biospin MRI GmbH, Ettlingen, Germany) on anesthetized mice. A 1.8 × 1.8 × 1.0 mm^3^ volume-of-interest was localized in the frontal cortex or 1.8 × 1.8 × 1.2 mm^3^ volume-of-interest was centered on the hippocampal formation. Water saturation was accomplished by means of three Gaussian-shaped CHESS RF pulses (90°–90°–180°).

Metabolite quantification involved spectral evaluation by LCModel (Provencher 1993) and calibration with brain water concentration (Duarte et al. 2014), for which the unsuppressed water proton signal served as an internal reference. The attenuation of the unsuppressed water signal was calibrated with *T*_2_ relaxation times of water protons in the volume-of-interest that was determined by a multi-echo spin-echo MRI (TR/TE = 2500/10–123 ms). *T*_1_ relaxation times were determined with the use of a spin-echo saturation recovery sequence and 7 TR values from 0.15 to 6 s. Metabolites with Cramer–Rao lower bounds above 20% were excluded from further analysis.

### Statistical analysis

Statistical evaluation was performed using SPSS^®^ (version 21.0, IBM^®^) and Microsoft Excel^®^. Alpha level (criterion of significance) was set to 0.05. Significant differences between two groups of data were determined by the Mann–Whitney’s *U* test.

## Results

### Magnetization transfer MRI delineates noradrenergic neuron groups based on proton-density contrast while saturating long-*T*_1_ extracellular protons

**Fig. 1:**
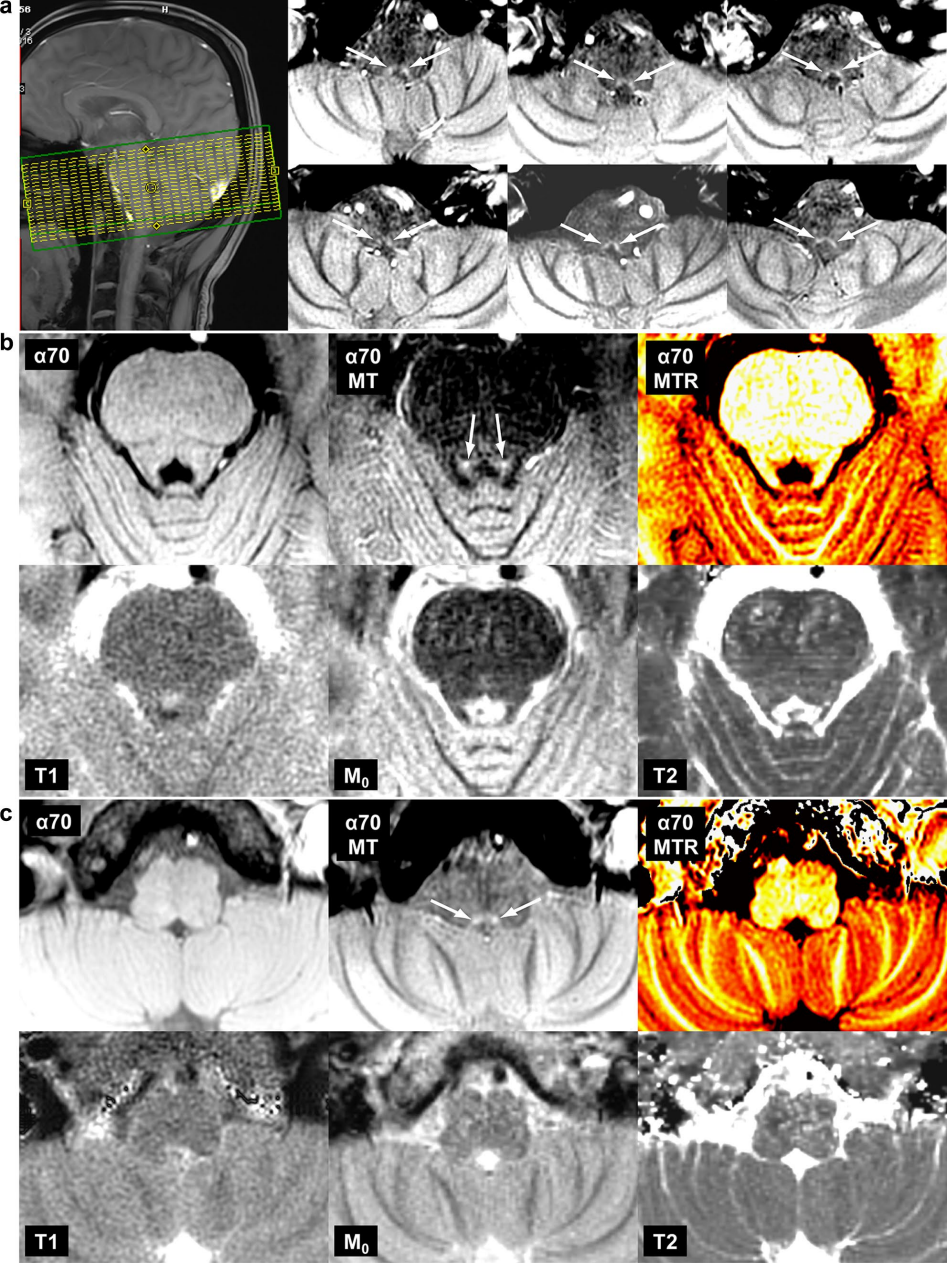
MRI delineates noradrenergic neuron groups in human brain in vivo. **a** (Left) Mid-sagittal human brain MRI illustrating the field-of-view selected for imaging the locus coeruleus and A2. (Right) Transversal MRI of A2 cell groups (arrows) in six different subjects. **b** Transversal *T*_1_-weighted MRI without (*α*70) and with MT (*α*70MT), MT ratio map (*α*70MTR), *T*_1_ map (*T*_1_), *M*_0_ map (*M*_0_), and *T*_2_ map (*T*_2_) showing the locus coeruleus (arrows) and **c** the A2 cell group (arrows)

**Fig. 2:**
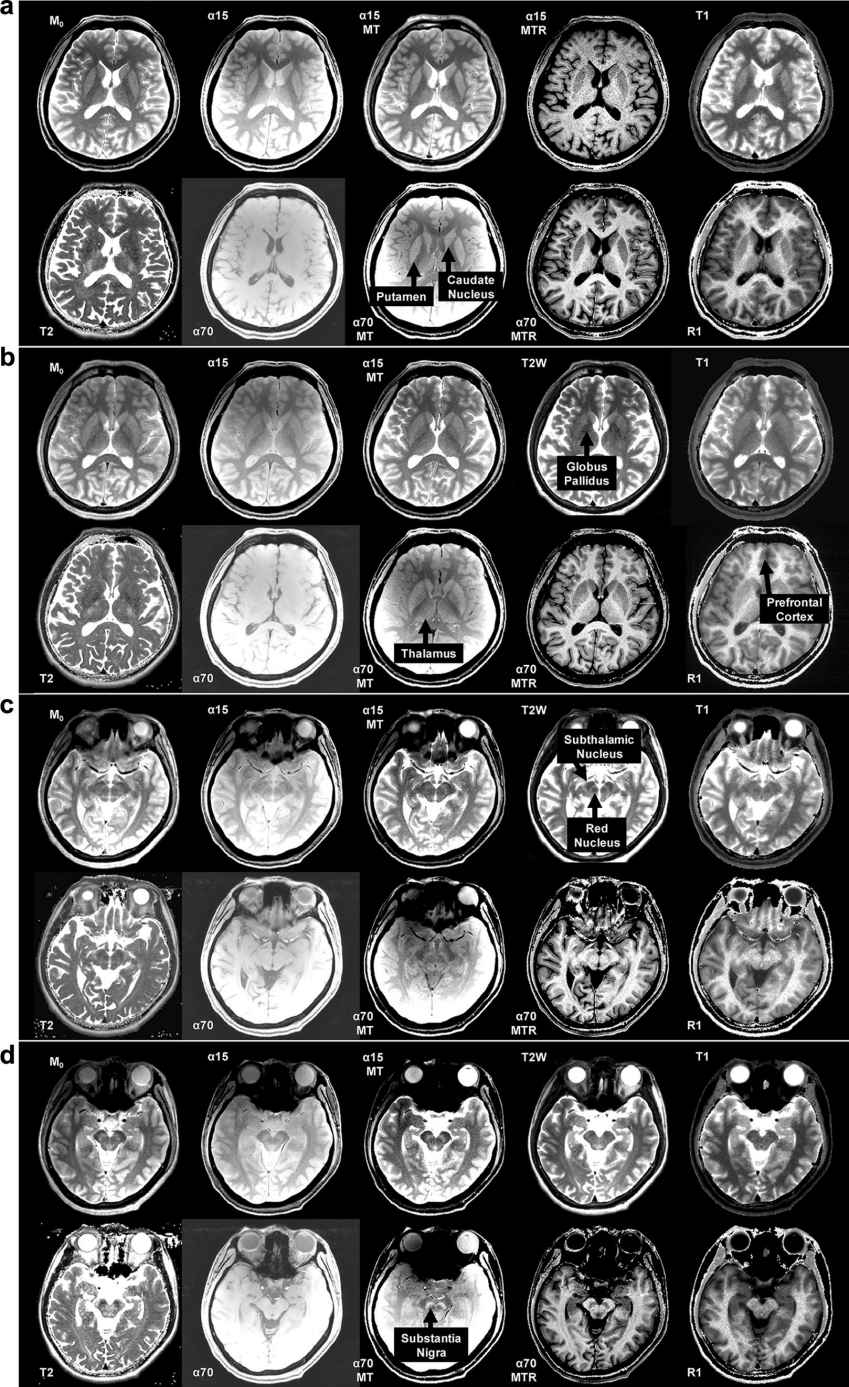
Magnetization transfer MRI provides proton-density contrast in brain while saturating long-*T*_1_ extracellular protons. **a** Transversal *M*_0_ map (*M*_0_), proton-density-weighted MRI without (*α*15) and with MT (*α*15 MT), MT ratio map (*α*15 MTR), *T*_1_ map (*T*_1_), *T*_2_ map (*T*_2_), *T*_1_-weighted MRI without (*α*70) and with MT (*α*70 MT), MT ratio map (*α*70 MTR), and *R*_1_ map (*R*_1_) showing the caudate nucleus and putamen, **b** globus pallidus, thalamus, and prefrontal cortex, **c** subthalamic and red nuclei, and **d** substantia nigra. Note the similar contrast for *M*_0,_*α*15 MT, and *α*70 MT except for cerebrospinal fluid

In human brainstem, MT-MRI delineated assemblies of NA neurons (Fig. 1). Figure 2 compares various quantitative maps of the human brain. The *M*_0_ map (*M*_0_), proton-density-weighted MRI with MT (*α*15 MT), and *T*_1_-weighted MRI with MT (*α*70 MT) show a similar contrast except for cerebrospinal fluid (CSF). The MT ratio maps (*α*15 MTR and *α*70 MTR) and *R*_1_ map (*R*_1_) also show a similar contrast. Quantitative evaluations (Supplementary Fig. 1, Table 1a) confirm these observations. Mean regional *R*_1_ of gray matter (GM) structures correlate linearly with water content (Supplementary Fig. 1a). The upward deviation of *R*_1_ of the WM from the linear relationship is attributable to its large interaction with water protons (Koenig et al. 1990). This effective cross-relaxation in WM is apparently cancelled out by the more pronounced spin-lock effect on GM structures, which results in an apparent linear relationship between the MT ratio (*α*70) and the water content including both GM and WM (Supplementary Fig. 1b). The values of the GM structures with significantly higher iron content, e.g., the globus pallidus and the red nucleus, and those with significantly higher copper content, e.g., the substantia nigra (SN) and LC, are well in line with those of other GM structures. Accordingly, regional signal intensities in *T*_1_-weighted MRI with MT (*α*70 MT) correlate significantly (*p* < .005) with those in *M*_0_ in every subject (Supplementary Fig. 1c), which also indicates that the regional spin density of intracellular water protons correlates significantly with the regional water content. Here, the NA cell groups were shown to have low MT ratios and high *T*_1_ and *T*_2_ values compared to other GM structures (Table 1). These findings indicate that the NA cell groups have high water content and that their highly concentrated copper is not the source of the high MRI signal intensity. For LC and A2 which are relatively unclear to delineate, the data were accumulated twice (TR/TE = 863/4.4 ms, total acquisition = 9 min 10 s) from another set of subjects that turned out to confirm the above findings (Table 2).

**Table 1:** Magnetization transfer ratios (2D FLASH, TR/TE = 863/4.4 ms, *α* = 70°), *T*_1_, and *T*_2_ of selected brain regions in human subjects (mean ± SD, *n* = 6, 31.5 ± 7.5 years old)

Brain regions	*α*70 MTR	*T*_1_ (s)	*T*_2_ (ms)
Frontal white matter	0.45 ± 0.01	0.74 ± 0.02	68.1 ± 2.3
Prefrontal cortex	0.34 ± 0.01	1.30 ± 0.13	96.0 ± 8.5
Caudate nucleus	0.36 ± 0.008	1.18 ± 0.05	76.4 ± 2.0
Putamen	0.38 ± 0.008	1.06 ± 0.03	67.6 ± 2.7
Thalamus	0.43 ± 0.02	0.95 ± 0.06	66.5 ± 8.0
Globus pallidus	0.43 ± 0.01	0.95 ± 0.12	64.7 ± 11
Red nuclues	0.47 ± 0.009	0.83 ± 0.04	65.5 ± 3.3
Subthalamic nucleus	0.45 ± 0.03	0.89 ± 0.05	60.3 ± 4.0
Substantia nigra	0.40 ± 0.01	0.99 ± 0.08	67.9 ± 2.5
Locus coeruleus	0.36 ± 0.008	1.18 ± 0.13	95.6 ± 4.2
Cerebrospinal fluid	0.03 ± 0.02	3.41 ± 0.25	409.0 ± 1.0

**Table 2:** Magnetization transfer ratios (2D FLASH, TR/TE = 863/4.4 ms, *α* = 70°), *T*_1_, and *T*_2_ of noradrenergic neuron groups in human subjects (mean ± SD, *n* = 6, 28.5 ± 2.3 years old)

Brain regions	*α*70 MTR	*T*_1_ (s)	*T*_2_ (ms)
Locus coeruleus	0.34 ± 0.02	1.02 ± 0.09	91.1 ± 9.6
A2	0.34 ± 0.03	1.16 ± 0.15	90.4 ± 10

**Fig. 3:**
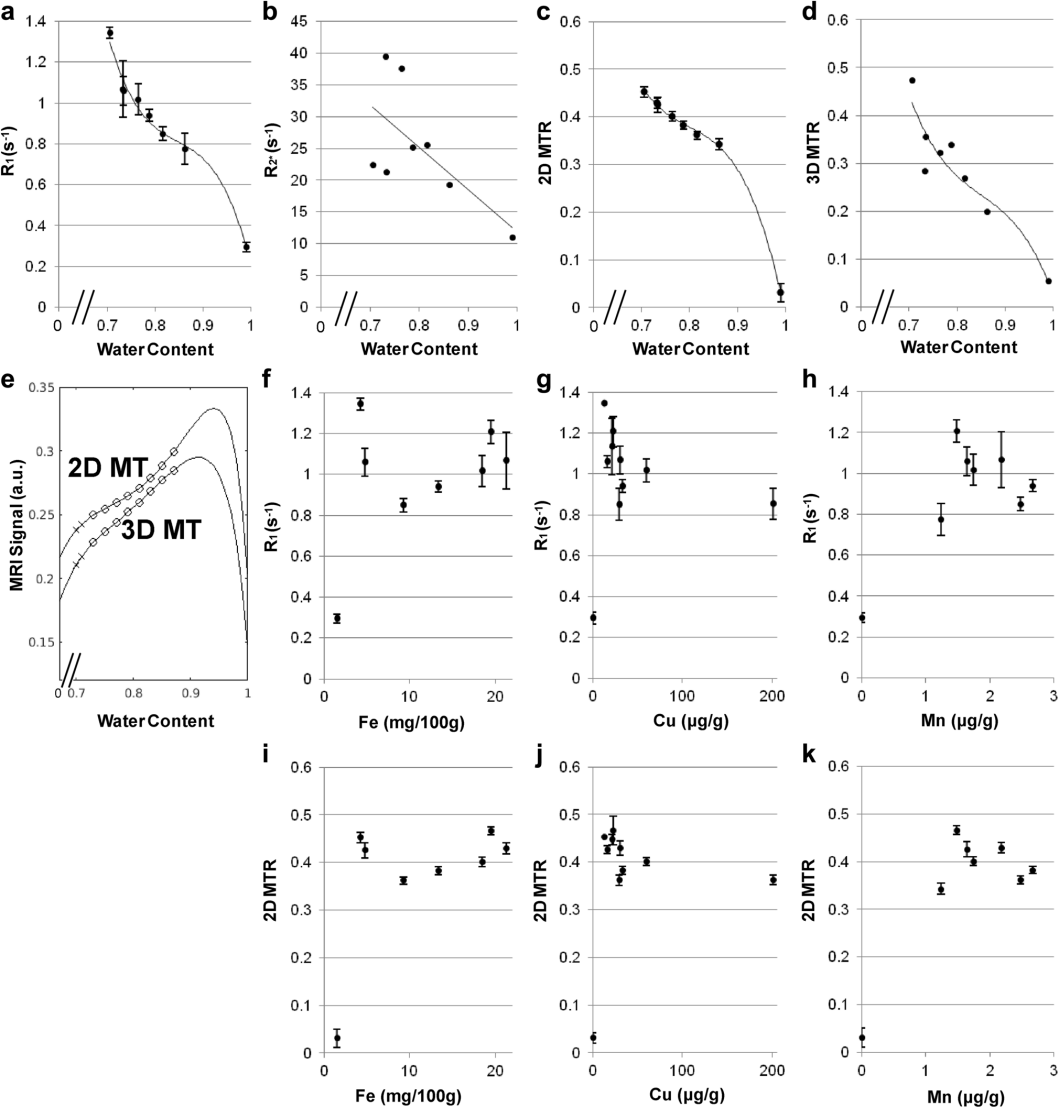
Magnetization transfer MRI provides proton-density contrast in brain while saturating long-*T*_1_ extracellular protons. **a***R*_1_, **b**$R_{2}^{*}$, **c** magnetization transfer ratio for 2D FLASH (TR/TE = 863/4.4 ms, *α* = 70°), and **d** magnetization transfer ratio for 3D FLASH (TR/TE = 47/7.5 ms, *α* = 22°) as a function of the water content. Equations and correlation coefficients are: **a***y* = − 113.29*x*^3^ + 287.52*x*^2^ + 244.5*x* + 70.47, *r* = − 0.99, **b***y* = − 66.351*x* + 78.25, *r* = − 0.65, **c***y* = − 35.676*x*^3^ + 85.538*x*^2^ + 68.888*x* + 19.011, *r* < − 0.99, **d***y* = − 30.301*x*^3^ + 77.288*x*^2^ + 66.429*x* + 19.466, *r* = − 0.94. **e** Calculated signal intensities for spoiled gradient-echo MRI as a function of water content. For a selected combination of acquisition parameters *α*, TR, and TE for 2D MT and 3D MT, the variables *R*_1_, $R_{2}^{*}$, and MTR are obtained from the functions given above. white circle = gray matter structure, multiplication sign = WM = frontal white matter. **f***R*_1_ plotted vs. the non-haemin iron content (mg iron/100 g fresh weight), **g** vs. the copper content (µg/g dry weight), and **h** vs. the manganese content (µg/g dry weight) as well as **i** magnetization transfer ratio (2D FLASH, TR/TE = 863/4.4 ms, *α* = 70°) plotted vs. the non-haemin iron content, **j** copper content, and **k** manganese content

Figure 3a–d shows the variables *R*_1_, $R_{2}^{*}$, and MTR as a function of tissue water content, which can be used for the calculation of signal intensities in gradient-echo MRI (Fig. 3e) for a selected combination of acquisition parameters *α*, TR, and TE. A strong on-resonance irradiation saturates the long-*T*_1_ water protons in the extracellular fluid with a water content of 0.99 (Fig. 3e). Additional MT suppresses the MRI signal in proportion to the macromolecular content (i.e., in inverse proportion to the water content), while the saturation of the extracellular water protons is preserved. As a result, the MT effect dominates for brain tissue (water content < 0.9), while the *T*_1_ effect dominates for extracellular fluid (water content > 0.95). When saturating extracellular long-*T*_1_ protons, the significant correlation between the signal intensities in MT-MRI and the proton density (Supplementary Fig. 1c) indicates that the signal intensities reflect the spin density of intracellular water protons whose *T*_1_ is shortened. In contrast to tissue water content, paramagnetic ion concentrations correlate neither with *R*_1_ nor with MT ratios (Fig. 3f–k). These findings indicate that the regional water content determines both MT ratio and *R*_1_.

**Fig. 4:**
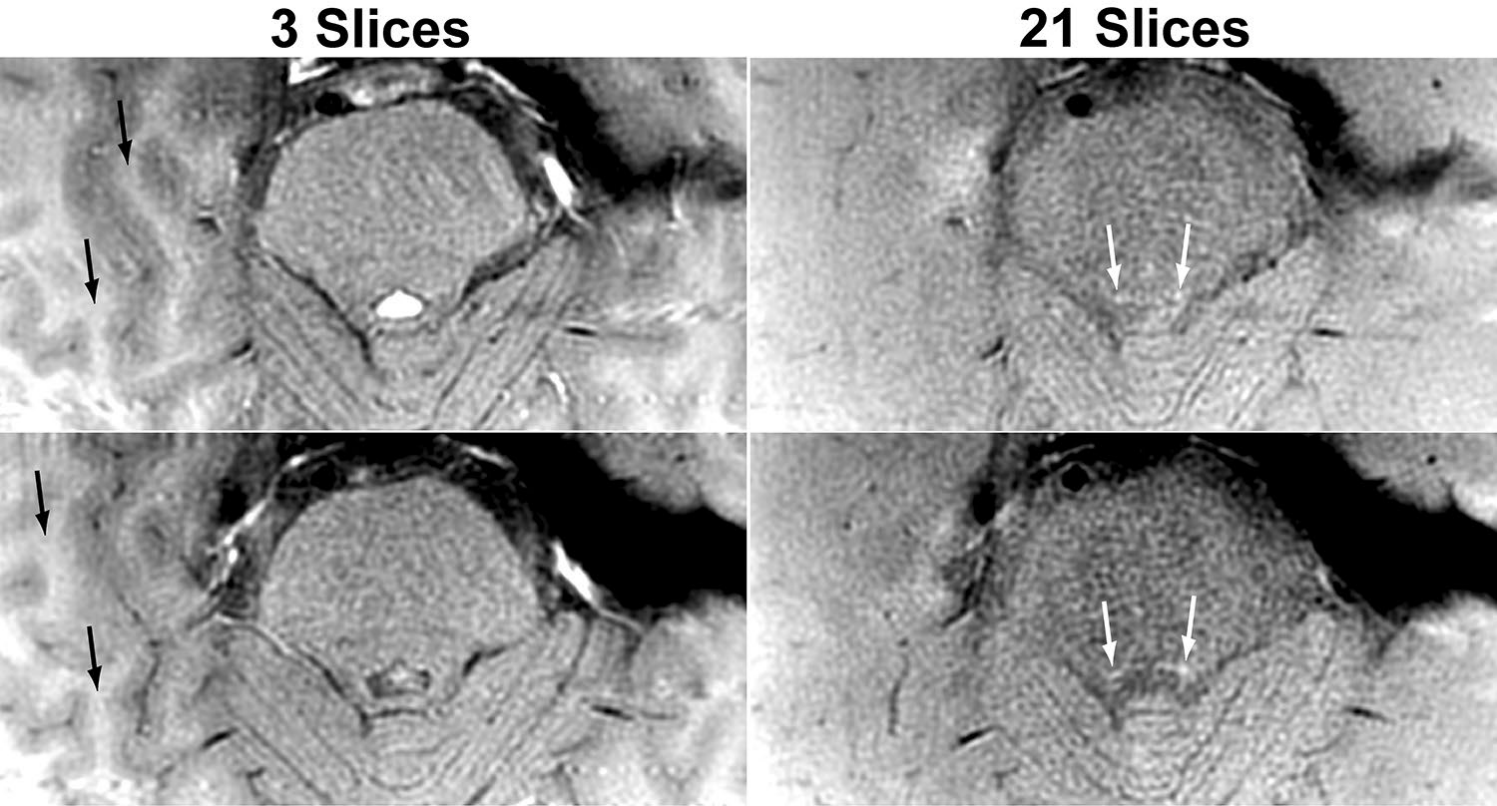
Locus coeruleus can be delineated by increasing the number of slices in a similar way as by specific off-resonance pulses. (Left column) Transversal 2D fast spin-echo MRI (TR/TE = 597/7.3 ms, flip angle = 150°) with the number of slices = 3 or (right column) 21 at two different levels of the locus coeruleus (white arrows) showing magnetization transfer effect induced by repetitive refocusing pulses. Also note that the signals of the subcortical white matter (black arrows) are predominantly suppressed by magnetization transfer effect in a 21-slice acquisition and, thus, its contrast to the cortex has disappeared or is even reversed

Figure 4 shows that LC can be delineated by increasing the number of slices in multi-slice 2D fast spin-echo MRI in a similar way as by additional MT pulses (cf., *α*70 and *α*70 MT in Fig. 1b). Although each slice is acquired in an interleaved manner, substantial MT effect is induced by the repetitive 150° refocusing pulses with frequency offset corresponding to location offset for each of the 21 slices.

### Magnetization transfer delineates noradrenergic neuron groups in the brainstem of mice in vivo

**Fig. 5:**
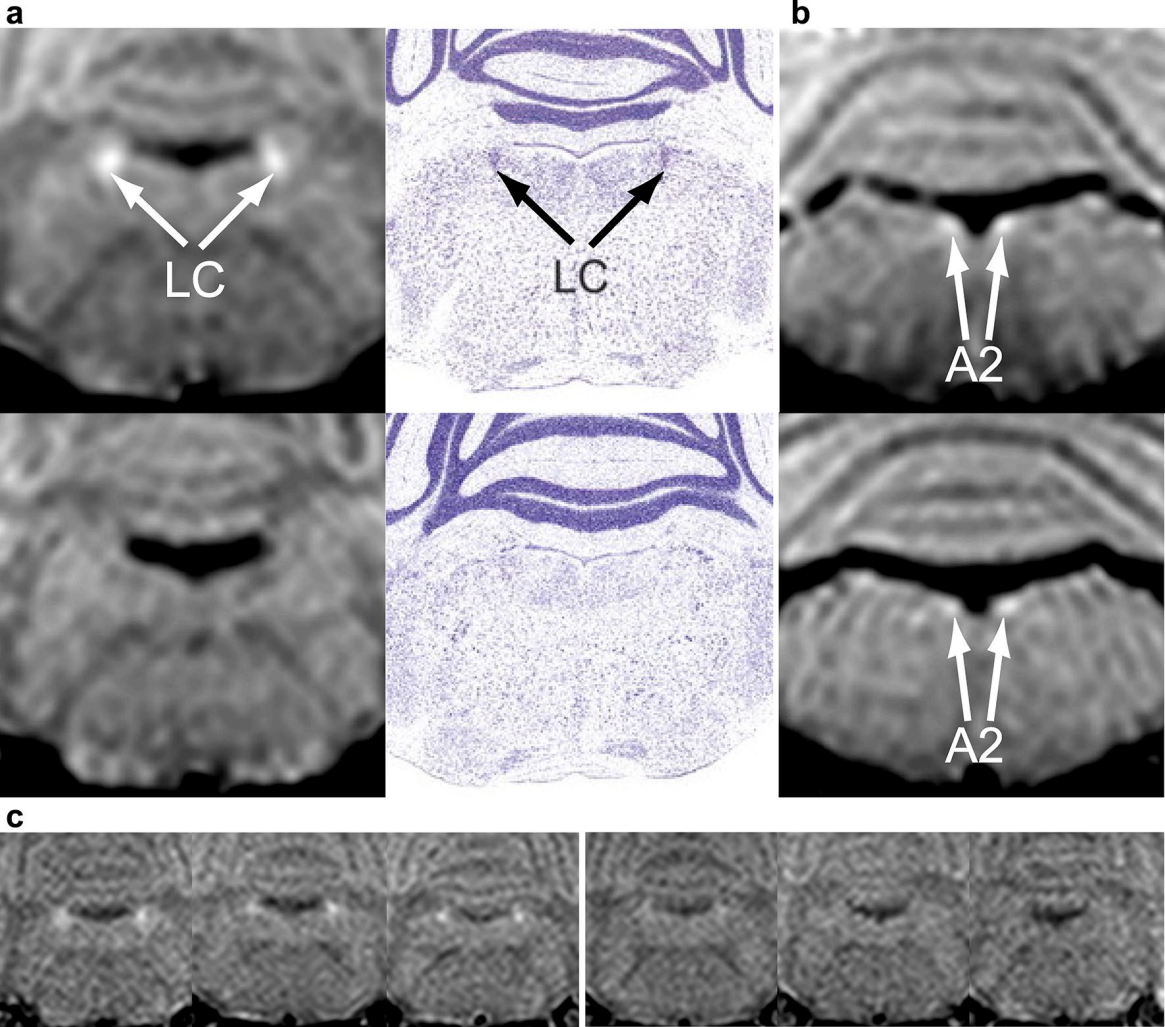
MRI delineates noradrenergic neuron groups in mouse brain in vivo. **a** (Left column) Coronal MRI (2.35 T, RF-spoiled 3D FLASH, TR/TE = 30/7.6 ms, *α* 22°, Δ*f* = 2500 Hz, *ω*_SAT_ = 523°/12 ms, 117 µm isotropic resolution) of the locus coeruleus of (top row) a 4-week-old female wild-type mouse and (bottom row) an Ear2(−/−) mouse in comparison with (right column) corresponding Nissl-stained sections (adapted from Warnecke et al. 2005). **b** (Top) coronal MRI sections of the A2 cell groups of a 4-week-old female wild-type mouse and (bottom) an Ear2(−/−) mouse. **c** (Left) Coronal MRI of the locus coeruleus of three different 4-week-old female wild-type mice and (right) Ear2(−/−) mice. The bright nuclei are only seen in wild-type mice and absent in mice with locus coeruleus neuron loss. *A2* A2 cell group, *LC* locus coeruleus

**Fig. 6:**
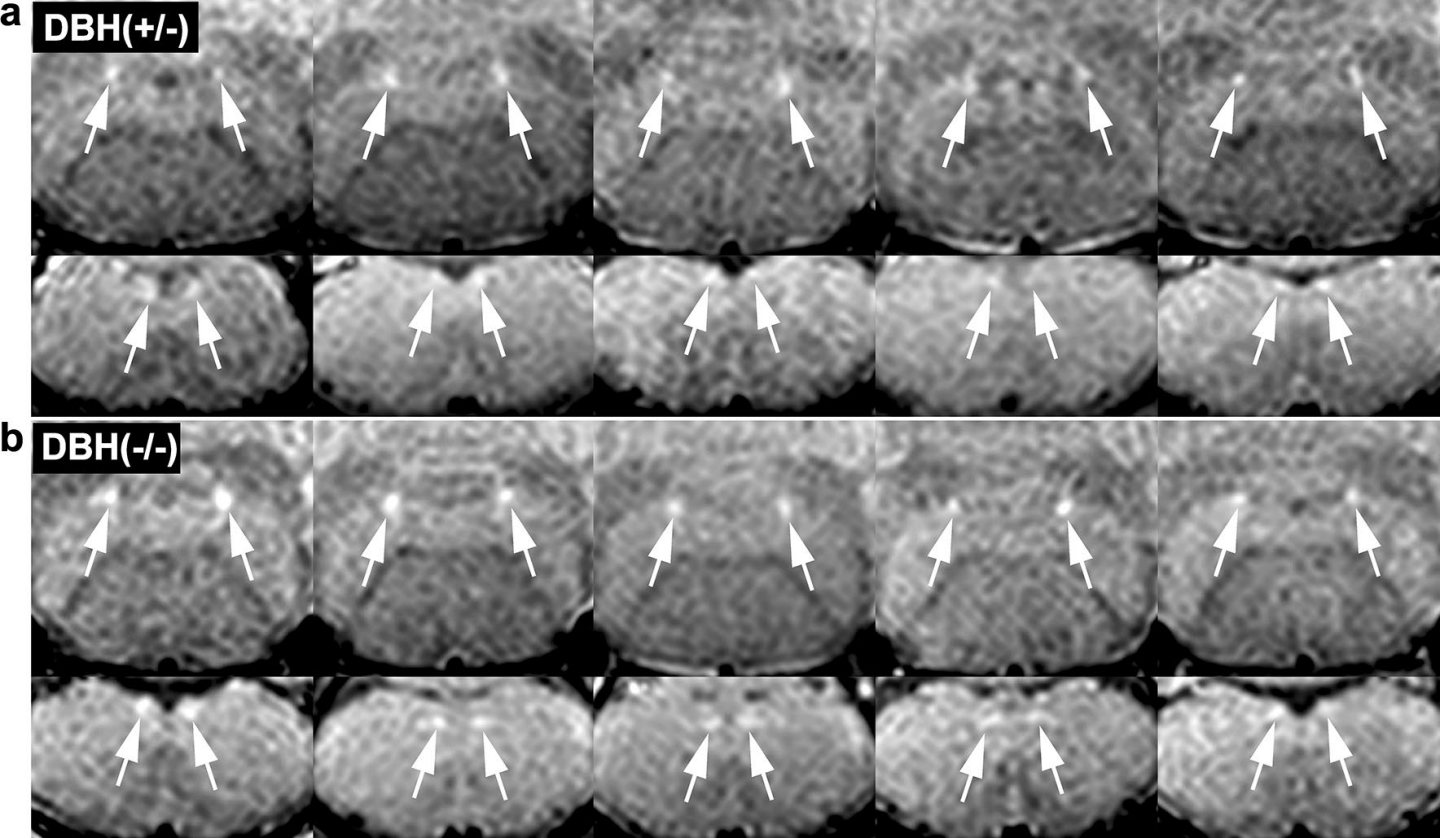
Dopamine β-hydroxylase has no effect on the MRI signal of the noradrenergic neurons. **a** Coronal MRI (for parameters, see Fig. 5) of the (upper row) locus coeruleus and (lower row) A2 cell groups of five 5-month-old control [DBH (+/−)] as well as of **b** five 5-month-old DBH (−/−) mice that lack dopamine β-hydroxylase

In the brainstem of mice, MRI delineated assemblies of NA neurons (Fig. 5). In young Ear2 (−/−) mice, MRI detected substantially less LC signal than in age- and gender-matched controls (Fig. 5, Supplementary Fig. 2) in agreement with the reduction in LC neuron numbers (Warnecke et al. 2005). Histologically, there was no neuromelanin (NM) observable in LC of control animals (Kummer et al. 2014; Warnecke et al. 2005) in agreement with previous reports (Barden and Levine 1983). This indicates that the NM is not the source of the high MRI signal intensity in mice. Figure 6 shows that dopamine β-hydroxylase has no influence on the MRI signals of NA cell groups. The SNR of LC is not significantly different in DBH(−/−) (47.9 ± 4.4, *n* = 5) compared with DBH(+/−) mice (46.3 ± 2.6, *n* = 5). This indicates that neither the presence of dopamine β-hydroxylase nor its binding to Cu^2+^ ions (Blumberg et al. 1965) is the source of the high MRI signal intensity.

**Fig. 7:**
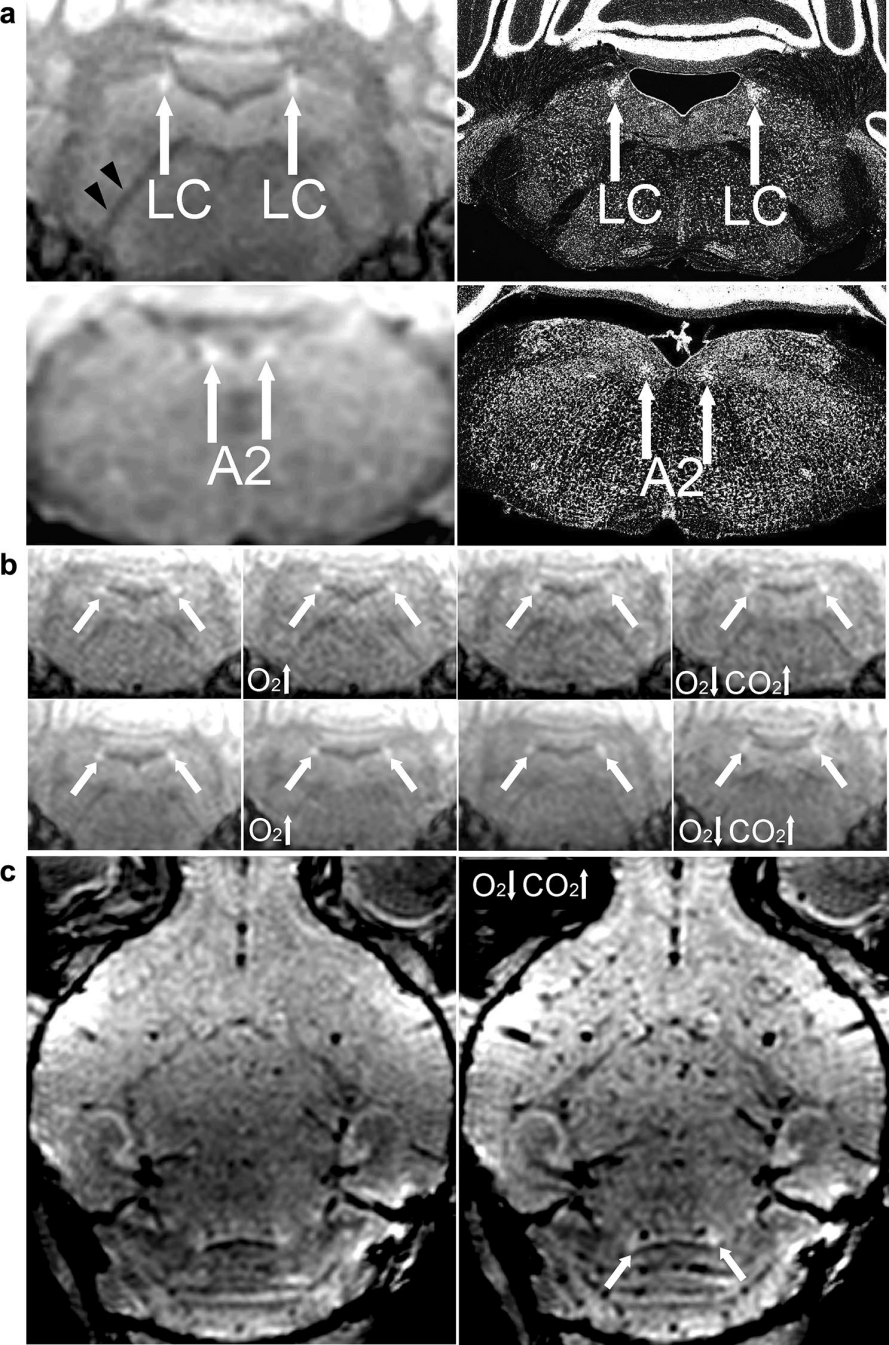
High signal intensity of the locus coeruleus is preserved during alterations in blood circulation. **a** High-resolution MRI of noradrenergic neurons in vivo at 9.4 T. (Left column) Coronal sections (80 µm isotropic resolution) in comparison with (right column) light microscopy of cell bodies (Nissl staining, contrast inverted, adapted from Mikula et al. 2007). LC = locus coeruleus, A2 = noradrenergic cell group 2 or dorsal motor nucleus of vagus, black arrowheads = white matter. **b** Coronal MRI (117 µm isotropic resolution) of 3-week-old male (top) animal no. 1 and (bottom) animal no. 2 under (from left to right) ketamine anesthesia, hyperoxia, 2% isoflurane, and hypoxic hypercapnia. Neither of these conditions that induce alterations in deoxyhemoglobin or blood flow substantially influences the signal intensity of the locus coeruleus (white arrows). **c** (Left) Horizontal MRI of animal no. 2 without and (right) with hypoxic hypercapnia. Note the preserved high signal intensity of the locus coeruleus (white arrows) despite the pronounced signal loss caused by increased deoxyhemoglobin content in dilated vasculature throughout the brain

Figure 7a confirms that MRI of NA neurons in vivo at higher spatial resolution and higher magnetic field is in agreement with light microscopy of cell bodies. Figure 7b, c shows that alterations in tissue oxygenation or perfusion do not substantially influence the signal intensity of LC. Increased deoxyhemoglobin content induced by hypoxic hypercapnia clearly accelerates $T_{2}^{*}$-dephasing (Fig. 7c) in brain tissue associated with dilated vasculature, whereas the high signal intensity of LC is barely affected. These findings indicate that the main source of the high signal intensity of NA neurons is neither presence nor inflow of hemoglobin, water, or other component of blood.

### Magnetization transfer MRI detects a noradrenergic neuron loss in a transgenic model of Alzheimer’s disease

**Fig. 8:**
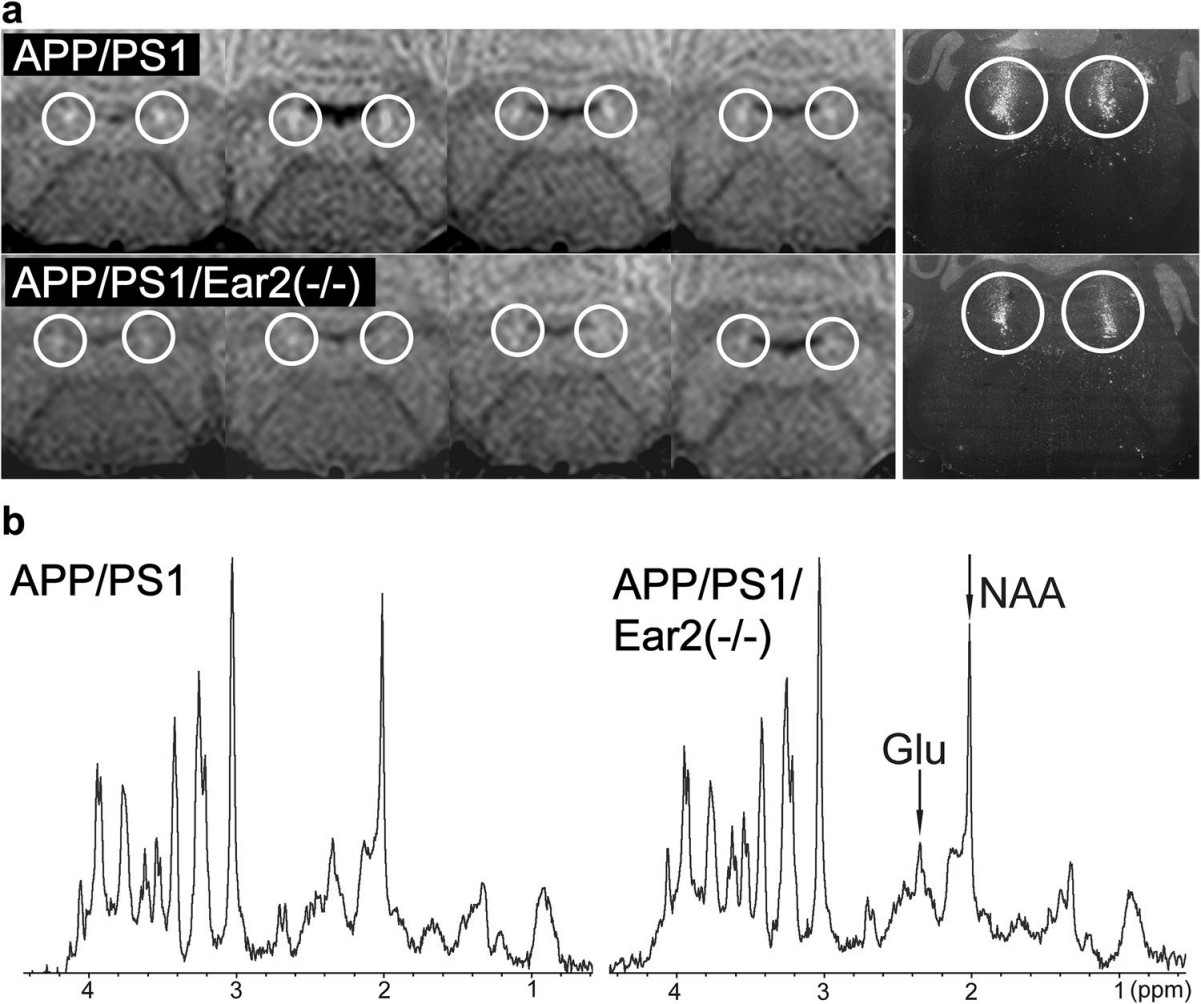
Magnetization transfer MRI detects a neuron loss in the locus coeruleus in a transgenic model of Alzheimer’s disease. **a** (Left) Coronal MRI (for parameters see Fig. 5) of the locus coeruleus of four different (top) APP/PS1 mice (19.8 ± 1.5 months) and (bottom) APP/PS1/Ear2(−/−) mice (20.0 ± 1.2 months) in comparison with (right) corresponding tyrosine hydroxylase in situ hybridization (adapted from Kummer et al. 2014, courtesy of Dr. Markus Kummer, contrast inverted). **b** Proton MRS (1.8 × 1.8 × 1.2 mm^3^ of the hippocampal formation) of (left) APP/PS1 (*n* = 8, 18.8 ± 5.8 months) and (right) APP/PS1/Ear2(−/−) (*n* = 10, 18.8 ± 3.6 months) mice in vivo (see Table 2). *Glu* glutamate, *NAA N*-acetylaspartate, *↓* significant decrease

In APP/PS1/Ear2(−/−) mice, a transgenic model of Alzheimer’s disease, MRI detected a reduced MRI signal in LC (Fig. 8a). The SNR of LC is significantly (*p* < .05) lower in APP/PS1/Ear2(−/−) (45.4 ± 5.3, *n* = 5, 19.6 ± 1.3 months old) than in APP/PS1 mice (57.1 ± 2.0, *n* = 4, 19.8 ± 1.5 months old) in agreement with a reduced number of LC neurons (Kummer et al. 2014). The LC neuron loss is associated with a 60–75% reduction of NA levels in projection areas, e.g., frontal cortex and hippocampus in 4- and 12-month-old APP/PS1/Ear2(−/−) mice (Kummer et al. 2014). Localized MRS revealed significant alterations of several metabolite concentrations in the brain of APP/PS1/Ear2(−/−) mice in vivo (Table 3; Supplementary Tables 1, 2; Supplementary Figs. 3, 4). These results are in line with previous MRS findings in human Alzheimer’s disease (Shonk et al. 1995). The MRS profile (Table 3) suggests (1) an impaired cellular respiration compensated for by accelerated anaerobic glycolysis (i.e., elevated lactate), (2) a loss of neurons (reduced *N*-acetylaspartate, glutamate, total creatine, and γ-aminobutyric acid) possibly compensated for by osmoregulators (elevated myo-inositol and taurine), (3) an accumulation of paramagnetic iron (shortened *T*_2_) associated with inflammation (Watanabe et al. 2016b), and (4) subsequent gliosis (elevated myo-inositol). More specifically, the chronic NA shortage caused by the LC neuron loss [APP/PS1 vs. APP/PS1/Ear2(−/−)] is responsible for the reduction of *N*-acetylaspartate (9.5 ± 1.9 vs. 7.4 ± 0.9 mM, *p* < .01) and glutamate (11.9 ± 1.7 vs. 9.4 ± 1.4 mM, *p* < .01) in the hippocampus as well as for the water proton *T*_2_-shortening (39.0 ± 1.1 vs. 37.8 ± 0.7 ms, *p* < .05) in the frontal cortex (Fig. 8b; Table 4). These MRS findings are in agreement with impaired spatial memory, disturbed synaptic plasticity, and increased astrogliosis as reported earlier (Kummer et al. 2014). The reduced glutamate concentration is in line with the assumption of positive feedback loops between NA and glutamate (Berridge and Waterhouse 2003).

**Table 3:** Concentration (mM) and concentration ratio of major cerebral metabolites as well as *T*_1_ and *T*_2_ relaxation times of water protons in APP/PS1/Ear2(−/−) and wild-type control mice

Genotype	Frontal cortex		Hippocampus	
	Wild type	APP/PS1/Ear2(−/−)	Wild type	APP/PS1/Ear2(−/−)
*n*: total (male)	*n* = 13 (4)	*n* = 11 (4)	*n* = 13 (4)	*n* = 11 (4)
Age (months)	20.5 ± 3.5	19.5 ± 4.1	20.5 ± 3.5	19.5 ± 4.1
tCr	11.2 ± 1.4	11.1 ± 1.3	13.0 ± 1.4	11.8 ± 0.8*
NAA	10.8 ± 0.6	9.9 ± 1.4*	9.0 ± 0.9	7.5 ± 0.9***
NAA + NAAG	12.0 ± 0.8	10.9 ± 1.3**	9.7 ± 1.1	8.4 ± 1.0**
Glu	13.5 ± 1.5	11.9 ± 2.1	11.4 ± 1.7	9.4 ± 1.3**
Lac	0.87 ± 0.6	2.1 ± 0.9**	1.2 ± 0.7	2.7 ± 1.3**
Ins	4.1 ± 0.7	5.3 ± 1.1*	5.3 ± 0.5	6.3 ± 1.6
Tau	10.4 ± 1.6	10.9 ± 1.3	12.2 ± 1.5	11.5 ± 1.0
GABA	2.1 ± 0.3	1.8 ± 0.4	2.4 ± 0.6	1.9 ± 0.4
GPC	1.3 ± 0.3	1.6 ± 0.5	1.5 ± 0.5	1.2 ± 0.4
GPC + PCh	2.2 ± 0.5	2.1 ± 0.3	2.2 ± 0.4	1.9 ± 0.5
NAA/tCr	0.98 ± 0.10	0.89 ± 0.11	0.69 ± 0.07	0.63 ± 0.08
NAA + NAAG/tCr	1.08 ± 0.13	0.99 ± 0.11	0.75 ± 0.08	0.72 ± 0.10
Glu/tCr	1.21 ± 0.07	1.07 ± 0.13**	0.87 ± 0.07	0.80 ± 0.11
Lac/tCr	0.08 ± 0.08	0.19 ± 0.07**	0.10 ± 0.05	0.23 ± 0.11**
Ins/tCr	0.37 ± 0.05	0.48 ± 0.11**	0.41 ± 0.04	0.53 ± 0.11**
Tau/tCr	0.93 ± 0.04	0.98 ± 0.09	0.94 ± 0.07	0.96 ± 0.07
GABA/tCr	0.19 ± 0.03	0.17 ± 0.03	0.19 ± 0.05	0.16 ± 0.03
GPC/tCr	0.12 ± 0.03	0.15 ± 0.05	0.11 ± 0.04	0.10 ± 0.04
GPC + PCh/tCr	0.19 ± 0.03	0.19 ± 0.03	0.17 ± 0.03	0.16 ± 0.04
*T*_1_ (s)	1.59 ± 0.12	1.59 ± 0.09	1.53 ± 0.09	1.53 ± 0.07
*T*_2_ (ms)	40.4 ± 2.1	37.8 ± 0.7**	41.5 ± 2.3	39.2 ± 1.3*

*GABA* γ-aminobutyric acid, *GPC* glycerophosphocholine, *Glu* glutamate, *Ins* myo-inositol, *Lac* lactate, *NAA N*-acetylaspartate, *NAAG N*-acetylaspartylglutamate, *PCh* phosphocholine, *Tau* taurine, *tCr* creatine + phosphocreatine

*p < .05, **p < .01, ***p < .001 vs. wild type in Mann–Whitney’s *U* test

**Table 4:** Concentration (mM) and concentration ratio of major cerebral metabolites as well as *T*_1_ and *T*_2_ relaxation times of water protons in APP/PS1/Ear2(−/−) compared to APP/PS1 mice

Genotype	Frontal cortex		Hippocampus	
	APP/PS1	APP/PS1/Ear2(−/−)	APP/PS1	APP/PS1/Ear2(−/−)
*n*: total (male)	*n* = 8 (4)	*n* = 10 (4)	*n* = 8 (4)	*n* = 10 (4)
Age (months)	18.8 ± 5.8	18.8 ± 3.6	18.8 ± 5.8	18.8 ± 3.6
tCr	12.0 ± 1.6	11.0 ± 1.0	12.0 ± 1.5	11.7 ± 0.7
NAA	10.6 ± 1.2	9.7 ± 1.2	9.5 ± 1.9	7.4 ± 0.9**
NAA + NAAG	11.7 ± 1.5	10.7 ± 1.1	10.6 ± 2.1	8.4 ± 1.0**
Glu	13.1 ± 1.6	11.8 ± 2.0	11.9 ± 1.7	9.4 ± 1.4**
Lac	1.8 ± 0.8	2.1 ± 0.9	1.7 ± 1.1	2.5 ± 1.1
Ins	5.1 ± 1.3	5.1 ± 1.2	5.6 ± 1.3	6.2 ± 1.4
Tau	11.1 ± 1.3	10.7 ± 1.4	11.3 ± 1.3	11.4 ± 0.9
GABA	1.9 ± 0.5	1.8 ± 0.4	2.1 ± 0.4	2.0 ± 0.4
GPC	1.4 ± 0.5	1.6 ± 0.5	1.5 ± 0.4	1.2 ± 0.5
GPC + PCh	2.0 ± 0.3	2.2 ± 0.3	1.9 ± 0.5	2.1 ± 0.4
NAA/tCr	0.89 ± 0.08	0.89 ± 0.12	0.80 ± 0.15	0.63 ± 0.09*
NAA + NAAG/tCr	0.96 ± 0.08	1.0 ± 0.09	0.89 ± 0.15	0.72 ± 0.10*
Glu/tCr	1.10 ± 0.11	1.08 ± 0.14	1.0 ± 0.12	0.79 ± 0.12**
Lac/tCr	0.15 ± 0.06	0.19 ± 0.07	0.15 ± 0.09	0.24 ± 0.11
Ins/tCr	0.42 ± 0.07	0.46 ± 0.09	0.46 ± 0.08	0.54 ± 0.11
Tau/tCr	0.93 ± 0.08	0.97 ± 0.09	0.94 ± 0.07	0.99 ± 0.07
GABA/tCr	0.16 ± 0.05	0.17 ± 0.03	0.18 ± 0.05	0.17 ± 0.03
GPC/tCr	0.12 ± 0.04	0.14 ± 0.05	0.13 ± 0.04	0.10 ± 0.04
GPC + PCh/tCr	0.19 ± 0.04	0.18 ± 0.02	0.16 ± 0.04	0.17 ± 0.02
*T*_1_ (s)	1.52 ± 0.08	1.58 ± 0.09	1.48 ± 0.06	1.52 ± 0.06
*T*_2_ (ms)	39.0 ± 1.1	37.8 ± 0.7*	40.5 ± 1.9	39.3 ± 1.4

*p < .05, **p < .01 vs. APP/PS1 in Mann–Whitney’s *U* test. For abbreviations see Table 3

## Discussion

### Magnetization transfer generates proton-density contrast in “*T*_1_-weighted” MRI of the brain

In principle, signal intensity in spoiled gradient-echo MRI reflects the spin density, *T*_1_, and $T_{2}^{*}$ of water protons. In *T*_1_-weighted MRI, repetitive strong on-resonance irradiation saturates the extracellular long-*T*_1_ protons while providing intracellular water protons with high signals. With additional MT, image contrast now primarily reflects the proton density (cf., equations in “Calculation of signal intensity in gradient-echo MRI of the brain”) and only secondarily the *T*_1_ relaxation (and $T_{2}^{*}$ even less). This is because the water protons whose *T*_1_ is shortened by diamagnetic molecules are proportionally saturated by the off-resonance irradiation **(**Supplementary Fig. 1a, b). The proton density effect dominates the observable signal for intracellular water (the water content of brain cells < 0.9), while the *T*_1_ effect applies to extracellular water (the water content of cerebrospinal fluid > 0.95) (Fig. 3e).

As a result, apart from long-*T*_1_ protons of extracellular water, additional MT actually turns “*T*_1_-weighted” MRI into “proton-density-weighted” MRI as far as the brain tissue itself is concerned. Here, “*T*_1_-weighted” applies only for the contrast between CSF and brain tissue itself. In other words, signal intensity in gradient-echo MRI with strong on-resonance and off-resonance irradiation reflects the density of water protons whose *T*_1_ is shortened by paramagnetic ions because the *T*_1_-shortening effect induced by diamagnetic molecules is cancelled by MT, whereas the *T*_1_-shortening effect induced by paramagnetic ions is not. For example, SN appears brighter than the red nucleus or cerebral peduncles in *α*15 and *α*70 images in Fig. 2 because of higher proton density in SN. The tissue surrounding SN contains water molecules bound to diamagnetic macromolecules where MT suppresses signal (more of them compared to SN).

So far, a number of studies reported the delineation of SN and LC by multi-slice 2D fast spin-echo MRI (Sasaki et al. 2006; Clewett et al. 2016). As shown in the present study (Figs. 1b, 4), the mechanism for contrast generation is basically the same for MT and for increasing the number of slices in 2D MRI with strong refocusing pulses: $T_{2}^{*}$ or *T*_2_ effect, respectively, is dominated by other factors, e.g., proton density. In this regard, spin-echo MRI is theoretically less efficient for delineating SN or LC than gradient-echo MRI because the time spent for refocusing hardly contributes to the delineation. Further, the MT effect generated by refocusing pulses is less controllable than the use of specific MT pulses because the MT effect depends on many other parameters, e.g., the number, direction, order, and gap of slice acquisition (Thomas et al. 2004). In more detail, the frequency offset of refocusing pulses is commonly smaller than that of a specific MT pulse, so that a more pronounced spin-lock effect is involved in “MT effect” by refocusing pulses. The generally higher *T*_1_/*T*_2_ ratios for cell assemblies than for WM or CSF in vivo (Table 1) indicate that the spin-lock effect is unfavorable for the delineation of cell assemblies from surrounding WM or CSF. Higher *T*_1_/*T*_2_ ratio results in a more pronounced spin-lock effect, i.e., lower signal intensities for the cell assemblies, whereas the lower *T*_1_/*T*_2_ ratio results in a less pronounced spin-lock effect, i.e., higher signal intensities for WM and CSF (cf., Supplementary Fig. 1a, b). Potential off-resonance artifacts in gradient-echo MRI may be minimized by increasing the spatial resolution.

The present study also showed that LC and A2 can be well delineated regardless of the field strength: the measurements of mice were carried out either at 2.35 or 9.4 T. In fact, this supports the view that the delineation of LC and A2 does not rely on *T*_1_ or *T*_2_ contrast but on the proton-density contrast because the relaxation times are field dependent.

### Paramagnetic ions in active cells

Every active cell needs paramagnetic ions as enzyme cofactors for electron transfer which serves essential processes (Que et al. 2008). In mammalian brain, these include cellular respiration, anti-oxidant defense, and catecholamine synthesis/metabolism, e.g., by electron transport chain, superoxide dismutase, tyrosine hydroxylase, and dopamine β-hydroxylase. As a consequence, metals such as iron, copper, and manganese, which possess a high capacity to gain and donate electrons, are the most abundant endogenous paramagnetic ions.

Most copper ions in brain are bound to proteins and thus their distribution among different sub-cellular compartments is tightly regulated (Lutsenko et al. 2010; Que et al. 2008). Therefore, the *T*_1_ relaxivity of Cu^2+^ in brain in vivo may be high compared to other paramagnetic ions, because Cu^2+^ has a long electron spin relaxation time (Burton et al. 1979). At 50 MHz, *T*_1_ relaxivity of free Cu^2+^ is about 0.5 L mmol^−1^ s^−1^ (Vymazal et al. 1998) that may be enhanced upon binding (Eisinger et al. 1961) to be about 5 L mmol^−1^ s^−1^ in vivo, whereas that of free Fe^3+^ is 6.9 L mmol^−1^ s^−1^ (Vymazal et al. 1998) that may be reduced (Eisinger et al. 1961) to about 3 L mmol^−1^ s^−1^. Given this favorable property of efficient *T*_1_-shortening, it is possible that the exceptionally high copper concentration plays a role in MT-MRI signal intensity of NA neurons, although the present study shows that it is the water proton density that plays a dominant role.

### Neuromelanin

Similar MRI contrast described for LC in human so far has been attributed to a water proton *T*_1_-shortening induced by NM (Sasaki et al. 2006; Clewett et al. 2016). NM bound to iron in vitro was shown to shorten *T*_1_ and thus to reduce MT ratio (Trujillo et al. 2017). However, it is unlikely that the MT contrast of specific cellular assemblies shown in the present study is due to unique *T*_1_-shortening molecules like NM. Firstly, if the high signal intensity of those structures (including LC) was due to the presence of *T*_1_-shortening molecules, then the *T*_1_ of those structures would have been shorter than other gray matter or surrounding structures. The present study showed that exactly the opposite is the case (Table 1). In fact, *T*_1_ relaxation times of some GM structures that contain no NM (e.g., thalamus, globus pallidus, red nucleus, and subthalamic nucleus) are even shorter than those of SN, LC, or A2 (Table 1) in line with a recent report (Priovoulos et al. 2018). If the high signal of SN and LC resulted from the *T*_1_-shortening by NM, the GM structures with shorter *T*_1_ would have yielded higher intensities than SN and LC.

These apparent contradictions can be explained as follows based on the present data (Figs. 1, 2, 3; Table 1): it is not the *T*_1_ relaxation but the water proton density that predominantly determines the in vivo MT-MRI contrast within the brain (cf., “Magnetization transfer MRI delineates noradrenergic neuron groups based on proton-density contrast while saturating long-*T*_1_ extracellular protons” and “Magnetization transfer generates proton-density contrast in “*T*_1_-weighted” MRI of the brain”). The in vitro evidence of *T*_1_-shortening by NM (Trujillo et al. 2017) does not necessarily mean that the delineation of NM-containing structures by in vivo MT-MRI results from the *T*_1_-shortening. Theoretically (cf., equations in “Calculation of signal intensity in gradient-echo MRI of the brain”); *T*_1_-shortening plays a role, but signal intensity is dominated by *M*_0_. In this regard, low MT ratios and high *T*_1_ and *T*_2_ values of SN, LC, and A2 as well as of caudate nucleus and putamen (Table 1) indicate that these structures have high water content and thus yield high signal intensities in MT-MRI (Figs. 1b, c, 2a). Conversely, high MT ratios and low *T*_1_ and *T*_2_ values of thalamus, globus pallidus, red nucleus, and subthalamic nucleus (Table 1) indicate that these structures have low water content and thus yield low signal intensities in MT-MRI (Fig. 2b, c).

Secondly, the present study demonstrated high MRI signal intensities in LC and A2 of 3- and 4-week-old mice (Figs. 5a–c, 7b, c). In SN of 15- to 24-month-old rats, electron microscopy revealed a few small NM granules in only 5% of SN neurons (DeMattei et al. 1986) while NM content increases with age. Another study showed that chronic exposure of rats to continuous florescent light increased NM deposition in SN (Romeo et al. 2013). However, given the lack of NM reported for NA neurons in rodents or for neurons in young rodents (Barden and Levine 1983), the high signals of LC and A2 in 4-week-old mice are not attributable to NM. In fact, although LC and A2 in the same kind of animals as those used in the present study have been intensively examined by a variety of histological techniques (Hammerschmidt et al. 2013; Kummer et al. 2014; Warnecke et al. 2005), the presence of NM, which could be readily detected under a simple light microscopy, has not been observed.

Thirdly, there is a substantial pool of paramagnetic ions in SN and LC that are not associated with NM (cf. “Paramagnetic ions in active cells”). Even when assuming that paramagnetic ions contributed to a brighter signal in *T*_1_-weighted images, the NM is unlikely to play a central role. The concentration of NM Fe in LC (SN) is only about 3.6 (20) ng/mg wet tissue, whereas the concentration of Fe in LC (SN) is as much as about 25 (150) ng/mg wet tissue (Zecca et al. 2004). The concentration of NM Cu in LC (SN) is only about 1.2 (0.4) ng/mg wet tissue, whereas the concentration of Cu in LC (SN) is as much as 31 (16) ng/mg wet tissue. In more detail, total Fe in LC of human is 5–45 ng/mg wet tissue. Fe bound to NM in LC is 1777 ± 92 ng/mg NM dry weight. NM concentration in LC is 1000–3200 ng/mg wet tissue. 1777 ng/mg NM dry weight multiplied by 1000–3200 ng/mg wet tissue yields 1.8–5.4 ng/mg wet tissue. Total Cu in LC is 8–64 ng/mg wet tissue. Cu bound to NM in LC is 605 ± 79 ng/mg of NM dry weight. 605 ng/mg NM dry weight multiplied by 1000–3200 ng/mg wet tissue yields 0.6–1.8 ng/mg wet tissue.

In general, NM as an effective metal chelator primarily serves to trap iron and provide neuronal protection from oxidative stress (Zucca et al. 2017). Excess dopamine or noradrenaline can be removed by converting it into a stable compound like NM, and this process rescues the cell. Thus, it must be noted that the present study using a mouse model of Alzheimer’s disease has to be cautiously translated into human medicine, because the amount and effect of NM may be different between mice and human.

### Blood flow and hemoglobin

MT is commonly used for magnetic resonance angiography to better delineate signal intensity in *T*_1_-weighted multi-slice 2D gradient-echo MRI caused by initial excitation of unsaturated blood water protons that have just flown into the imaging plane, i.e., time-of-flight effect. Here, in MRI of mouse brain, LC and A2 are well within (and off the margin of) the volume that is excited only as a 3D slab by a moderate flip angle of 22°. Thus, time-of-flight effect is not so pronounced as in 2D MRI. Further, high signal intensity is observed (not in vessels but) in nerve cell assemblies like LC and A2 which gain blood supply through capillary beds. In such brain tissue (e.g., the visual cortex), the signal intensity change was shown to be independent from the excitation flip angle, i.e., from the time-of-flight effect (Frahm et al. 1994).

By contrast, the signal intensity in the visual cortex was shown (Frahm et al. 1994) to be significantly dependent on brain activation (i.e., visual stimulation by light) that is associated with washout of $T_{2}^{*}$-shortening paramagnetic deoxyhemoglobin from capillary beds by inflowing diamagnetic oxyhemoglobin. Although the preserved high signal intensity of LC despite the pronounced signal loss caused by increased deoxyhemoglobin content in dilated vasculature (Fig. 7c) indicates that hemoglobin is not the main source of the high signal of NA neurons in the present study, the blood-oxygenation-level-dependent effect is theoretically expected and cannot be excluded for LC. Given the high exposure of LC neurons to blood circulation through dense capillaries (Mather and Harley 2016), the $T_{2}^{*}$ effect could have been well observed if the gradient-echo MRI was performed with a longer echo time. However, the present study sought for proton-density rather than blood-oxygenation-level-dependent contrast.

### Dopaminergic neurons

The present study shows that SN as well as LC yields high signal intensities in MT-MRI of human brain (*α*70MT in Fig. 2d) in agreement with a number of previous studies (Sasaki et al. 2006). This clear delineation is probably associated with the large size of the pars compacta in SN as well as with the tight packing of the large dopaminergic cells in the pars compacta. In primates, the pars compacta is as large as the rest of SN, the pars reticulata (Voogd 1998). By contrast, SN was not delineated in MT-MRI of mouse brain in the present study. This can be explained by partial volume effect because, in contrast to primates, the pars compacta is substantially smaller than the pars reticulata in rodents (Voogd 1998).

## Conclusions

To summarize, this is the first report about the in vivo MRI visibility of A2 cell assemblies in human as well as of NA neurons in animals. The absence of the high MRI signal from LC of Ear2(−/−) mice confirms the cell bodies of NA neurons as a source of the high signal. The preservation of the high signal of NA neurons in DBH(−/−) mice indicates that the source of the high signal is neither dopamine β-hydroxylase nor their binding to paramagnetic ions. The detection of these NA cell groups in young wild-type mice by *T*_1_-weighted MRI with MT, the reduced contrast in Ear2 (−/−) mice, and the preserved contrast in DBH (−/−) mice together indicate that the source of the high MRI signal is neither NM nor dopamine β-hydroxylase, but a high density of water protons whose *T*_1_ is shortened by paramagnetic ions. Application of this MRI method to a transgenic model of Alzheimer’s disease illustrated its potential use in neuroradiology. Given that the decline in LC neuron numbers is associated with aging, dementia, A*β* plaque load, and the progression of Alzheimer’s disease (Kummer et al. 2014), it is foreseeable that MRI of NA neurons will play an increasing role in translational biomedical research of neurodegenerative diseases.

## Electronic supplementary material

Below is the link to the electronic supplementary material.

Supplementary material 1 (DOCX 1489 KB)

## Abbreviations

A2: Noradrenergic neuron group 2
GM: Gray matter
LC: Locus coeruleus
MRS: Magnetic resonance spectroscopy
MT: Magnetization transfer
NA: Noradrenaline
NM: Neuromelanin
WM: White matter

## References

[CR1] Barden H, Levine S (1983). Histochemical observations on rodent brain melanin. Brain Res Bull.

[CR2] Berridge CW, Waterhouse BD (2003). The locus coeruleus–noradrenergic system: modulation of behavioral state and state-dependent cognitive processes. Brain Res Rev.

[CR3] Blumberg WE, Goldstein M, Lauber E, Peisach J (1965). Magnetic resonance studies on the mechanism of the enzymic β-hydroxylation of 3, 4-dihydroxyphenylethylamine. Biochim Biophys Acta.

[CR4] Burton DR, Forsen S, Karlstrom G, Dwek RA (1979). Proton relaxation enhancement (PRE) in biochemistry: a critical survey. Prog Nucl Magn Reson Spectrosc.

[CR5] Clewett DV, Lee TH, Greening S, Ponzio A, Margalit E, Mather M (2016). Neuromelanin marks the spot: identifying a locus coeruleus biomarker of cognitive reserve in healthy aging. Neurobiol Aging.

[CR6] Dahlström A, Fuxe K (1964). Evidence for the existence of monoamine-containing neurons in the central nervous system. Demonstration of monoamines in the cell bodies of brain stem neurons. Acta Physiol Scand Suppl.

[CR7] Deistung A, Schäfer A, Schweser F, Biedermann U, Turner R, Reichenbach JR (2013). Toward in vivo histology: a comparison of quantitative susceptibility mapping (QSM) with magnitude-, phase-, and *R*_2_*-imaging at ultra-high magnetic field strength. Neuroimage.

[CR8] DeMattei M, Levi AC, Fariello RG (1986). Neuromelanic pigment in substantia nigra neurons of rats and dogs. Neurosci Lett.

[CR9] Duarte JMN, Do KQ, Gruetter R (2014). Longitudinal neurochemical modifications in the aging mouse brain measured in vivo by ^1^H magnetic resonance spectroscopy. Neurobiol Aging.

[CR10] Duflou H, Maenhaut W, De Reuck J (1989). Regional distribution of potassium, calcium, and six trace elements in normal human brain. Neurochem Res.

[CR11] Eisinger J, Shulman RG, Blumberg WE (1961). Relaxation enhancement by paramagnetic ion binding in deoxyribonucleic acid solutions. Nature.

[CR12] Frahm J, Merboldt KD, Hänicke W, Kleinschmidt A, Boecker H (1994). Brain or vein—oxygenation or flow? On signal physiology in functional MRI of human brain activation. NMR Biomed.

[CR13] Gelman N, Ewing JR, Gorell JM, Spickler EM, Solomon EG (2001). Interregional variation of longitudinal relaxation rates in human brain at 3.0T: relation to estimated iron and water contents. Magn Reson Med.

[CR14] Hallgren B, Sourander P (1958). The effect of age on the non-haemin iron in the human brain. J Neurochem.

[CR15] Hammerschmidt T, Kummer MP, Terwel D, Martinez A, Gorji A, Pape HC, Rommelfanger KS, Schroeder JP, Stoll M, Schultze J, Weinshenker D, Heneka MT (2013). Selective loss of noradrenaline exacerbates early cognitive dysfunction and synaptic deficits in APP/PS1 mice. Biol Psychiatry.

[CR16] Heneka MT, Carson MJ, El Khoury J, Landreth GE, Brosseron F, Feinstein DL (2015). Neuroinflammation in Alzheimer’s disease. Lancet Neurol.

[CR17] Henkelman RM, Stanisz GJ, Graham SJ (2001). Magnetization transfer in MRI: a review. NMR Biomed.

[CR18] Jankowsky JL, Slunt HH, Ratovitski T, Jenkins NA, Copeland NG, Borchelt DR (2001). Co-expression of multiple transgenes in mouse CNS: a comparison of strategies. Biomol Eng.

[CR19] Kaufman SJ (1974). Dopamine-beta-hydroxylase. Psychiatry Res.

[CR20] Koenig SH, Brown RD, Spiller M, Lundbom N (1990). Relaxometry of brain: why white matter appears bright in MRI. Magn Reson Med.

[CR21] Kummer MP, Hammerschmidt T, Martinez A, Terwel D, Eichele G, Witten A, Figura S, Stoll M, Schwartz S, Pape HC, Schultze JL, Weinshenker D, Heneka MT (2014). Ear2 deletion causes early memory and learning deficits in APP/PS1 mice. J Neurosci.

[CR22] Lutsenko S, Bhattacharjee A, Hubbard AL (2010). Copper handling machinery of the brain. Metallomics.

[CR23] Mather M, Harley CW (2016). The locus coeruleus: essential for maintaining cognitive function and the aging brain. Trends Cogn Sci.

[CR24] Mikula S, Trotts I, Stone JM, Jones EG (2007). Internet-enabled high-resolution brain mapping and virtual microscopy. Neuroimage.

[CR25] Moore RY, Bloom FE (1978). Central catecholamine neuron systems: anatomy and physiology of the dopamine systems. Ann Rev Neurosci.

[CR26] Priovoulos N, Jacobs HI, Ivanov D, Uludağ K, Verhey FR, Poser BA (2018). High-resolution in vivo imaging of human locus coeruleus by magnetization transfer MRI at 3T and 7T. Neuroimage.

[CR27] Prohaska JR (1987). Functions of trace elements in brain metabolism. Physiol Rev.

[CR28] Provencher SW (1993). Estimation of metabolite concentrations from localized in vivo proton NMR spectra. Magn Reson Med.

[CR29] Que EL, Domaille DW, Chang CJ (2008). Metals in neurobiology: probing their chemistry and biology with molecular imaging. Chem Rev.

[CR30] Romeo S, Viaggi C, Di Camillo D, Willis AW, Lozzi L, Rocchi C (2013). Bright light exposure reduces TH-positive dopamine neurons: implications of light pollution in Parkinson’s disease epidemiology. Sci Rep.

[CR31] Sasaki M, Shibata E, Tohyama K, Takahashi J, Otsuka K, Tsuchiya K, Takahashi S, Ehara S, Terayama Y, Sakai A (2006). Neuromelanin magnetic resonance imaging of locus ceruleus and substantia nigra in Parkinson’s disease. Neuroreport.

[CR01] Schulz H, Johner C, Eder G, Ziesenis A, Reitmeier P, Heyder J, Balling R (2002). Respiratory mechanics in mice: strain and sex specific differences. Acta Physiol Scand.

[CR32] Shonk TK, Moats RA, Gifford P, Michaelis T, Mandigo JC, Izumi J, Ross BD (1995). Probable Alzheimer disease: diagnosis with proton MR spectroscopy. Radiology.

[CR33] Tammer R, Boretius S, Michaelis T, Pucher-Diehl A (2007) European patent no. 2,174,154, US patent no. 8,334,698 B2

[CR34] Tan Z (2016) Advances in real-time phase-contrast flow MRI and multi-echo radial FLASH. Dissertation. University of Göttingen, pp 73–76

[CR35] Thomas DL, De Vita E, Roberts S, Turner R, Yousry TA, Ordidge RJ (2004). High-resolution fast spin echo imaging of the human brain at 4.7 T: implementation and sequence characteristics. Magn Reson Med.

[CR36] Trujillo P, Summers PE, Ferrari E, Zucca FA, Sturini M, Mainardi LT (2017). Contrast mechanisms associated with neuromelanin-MRI. Mag Reson Med.

[CR37] Voogd J, Nieuwenhuys R, Ten Donkelaar HJ, Nicholson C (1998). MesencephalonThe central nervous system of vertebrates.

[CR38] Vymazal J, Brooks RA, Bulte JWM, Gordon D, Aisen P (1998). Iron uptake by ferritin: NMR relaxometry studies at low iron loads. J Inorg Biochem.

[CR39] Wang X, Roeloffs VB, Merboldt KD, Voit D, Schätz S, Frahm J (2015). Single-shot multi-slice *T*1 mapping at high spatial resolution—inversion-recovery FLASH with radial undersampling and iterative reconstruction. Open Med Imaging J.

[CR40] Wang X, Roeloffs V, Klosowski J, Tan Z, Voit D, Uecker M, Frahm J (2018). Model-based *T*1 mapping with sparsity constraints using single-shot inversion-recovery radial FLASH. Magn Reson Med.

[CR41] Wansapura JP, Holland SK, Dunn RS, Ball WS (1999). NMR relaxation times in the human brain at 3.0 T. J Magn Reson Imaging.

[CR42] Warnecke M, Oster H, Revelli JP, Alvarez-Bolado G, Eichele G (2005). Abnormal development of the locus coeruleus in Ear2 (Nr2f6)-deficient mice impairs the functionality of the forebrain clock and affects nociception. Genes Dev.

[CR43] Warren P, Earl C, Thompson R (1960). The distribution of copper in human brain. Brain.

[CR44] Watanabe T, Radulovic J, Spiess J, Natt O, Boretius S, Frahm J, Michaelis T (2004). In vivo 3D MRI staining of the mouse hippocampal system using intracerebral injection of MnCl_2_. Neuroimage.

[CR45] Watanabe T, Frahm J, Michaelis T (2012). Myelin mapping in the central nervous system of living mice using contrast-enhanced magnetization transfer MRI. Neuroimage.

[CR46] Watanabe T, Frahm J, Michaelis T (2016). Amide proton signals as pH indicator for in vivo MRS and MRI of the brain—responses to hypercapnia and hypothermia. Neuroimage.

[CR47] Watanabe T, Frahm J, Michaelis T (2016). In vivo brain MR imaging at subnanoliter resolution: contrast and histology. Mag. Reson Med Sci.

[CR48] Wood JH, Lajtha A (1982). Physiological neurochemistry of cerebrospinal fluid. Handbook of neurochemistry: chemical and cellular architecture.

[CR49] Yao B, Li TQ, van Gelderen P, Shmueli K, de Zwart JA, Duyn JH (2009). Susceptibility contrast in high field MRI of human brain as a function of tissue iron content. Neuroimage.

[CR50] Zecca L, Stroppolo A, Gatti A, Tampellini D, Toscani M, Gallorini M, Giaveri G, Arosio P, Santambrogio P, Fariello RG, Karatekin K, Kleinman MH, Turro N, Hornykiewicz O, Zucca FA (2004). The role of iron and copper molecules in the neuronal vulnerability of locus coeruleus and substantia nigra during aging. Proc Natl Acad Sci USA.

[CR51] Zucca FA, Segura-Aguilar J, Ferrari E, Muñoz P, Paris I, Sulzer D (2017). Interactions of iron, dopamine and neuromelanin pathways in brain aging and Parkinson’s disease. Prog Neurobiol.

