# Supplementary material for: Magnetic resonance imaging of noradrenergic neurons
Source: Brain Struct Funct. 2019 Mar 22;224(4):1609–25. doi: 10.1007/s00429-019-01858-0 (PMC6509075; doi:10.1007/s00429-019-01858-0)
Supplement: Supplementary file 1 — Supplementary material 1 (DOCX 1489 KB) [file 429_2019_1858_MOESM1_ESM.docx]

**
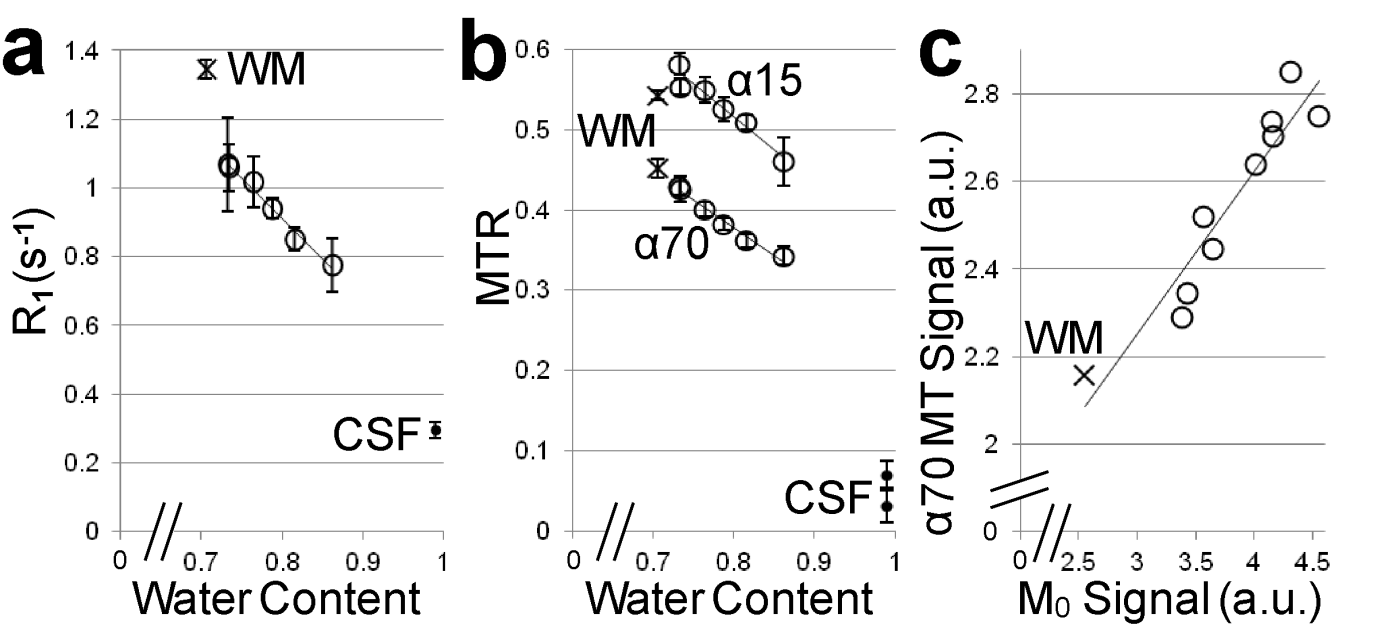
**

**Supplementary Figure 1** (a) R_1_ and (b) MT ratios (α15 MTR and α70 MTR) of various regions in the brain plotted vs. water content. Equations and correlation coefficients are: (a) y = -2.3556x + 2.796, r = -0.99, (b) y = -0.8181x + 1.1706, r = -0.97 for α15 and y = -0.6843x + 0.9263, r = -0.99 for α70. (c) Significant (r=0.94, p<.0005) correlation of regional signal intensities in α70 MT plotted vs. M_0_ (single subject).

**
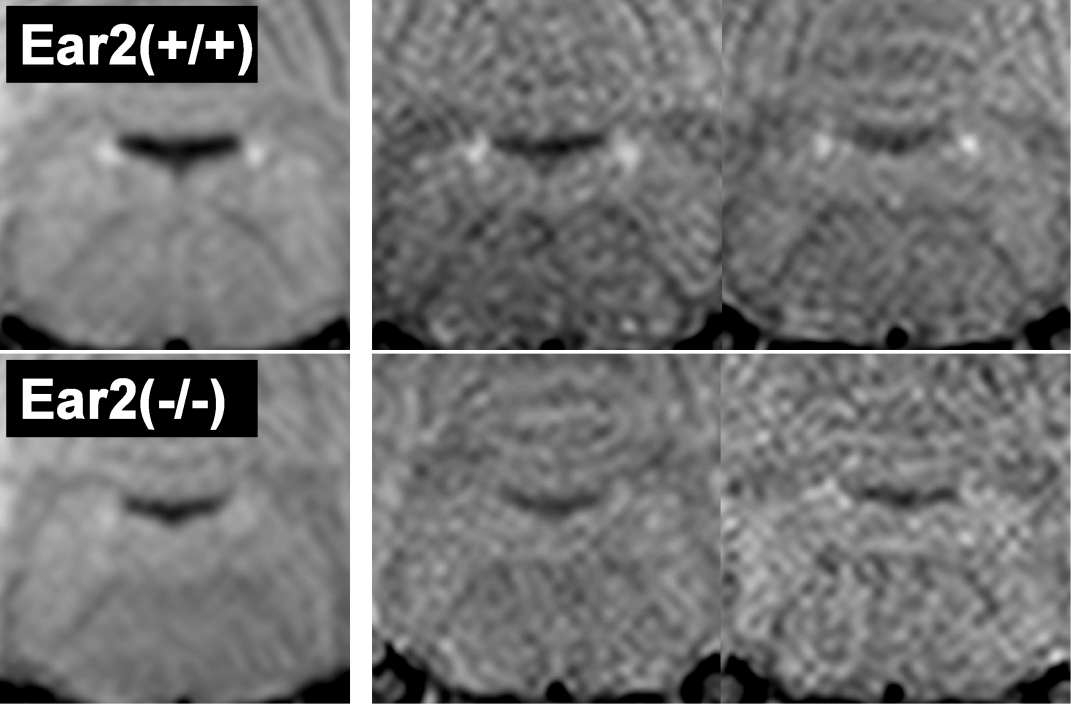
**

**Supplementary Figure 2** (First column) coronal MRI (α 22°, field-of-view = 30 × 18.75 × 22.5 mm^3^, matrix = 256 × 160 × 192, Δf = 2500 Hz, ω_SAT_ = 512°/12 ms) or (second and third column) (Δf = 5000 Hz, ω_SAT_ = 1024°/12 ms) of the locus coeruleus of 4-week-old male (upper row) wild-type and (bottom row) Ear2(-/-) mice.


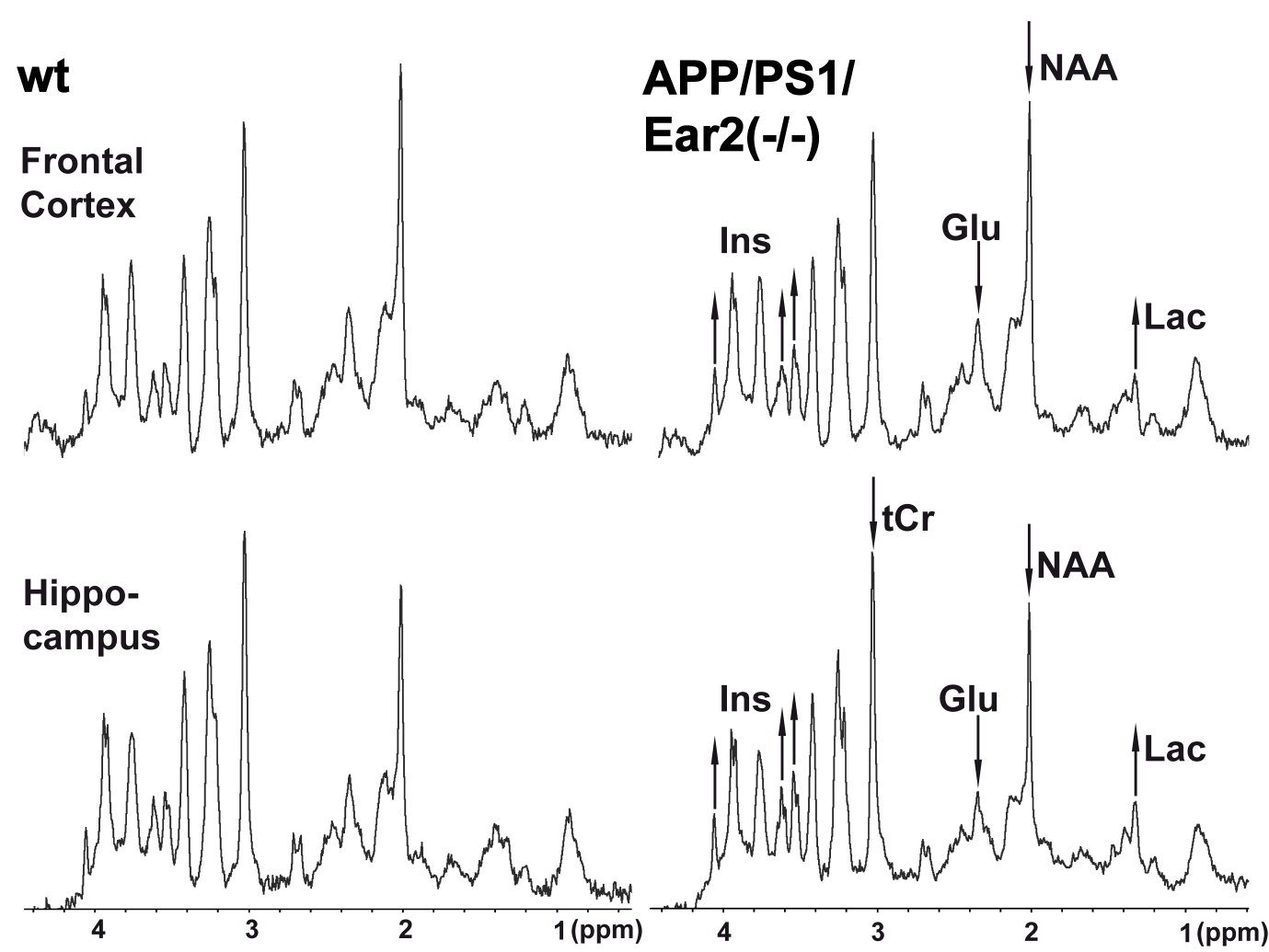


**Supplementary Figure 3** (Top row) MRS of the frontal cortex (STEAM, TR/TE/TM = 6000/10/10 ms, 768 averages, 1.8×1.8×1.0 mm^3^) and (bottom row) hippocampal formation (512 averages, 1.8×1.8×1.2 mm^3^) in (left column) wild-type (n=13) and (right column) APP/PS1/Ear2(-/-) mice *in vivo* (n=11). Exact values and ratios are summarized in Supplementary Table 4a. Glu = glutamate, Ins = myo-inositol, Lac = lactate, NAA = *N*-acetylaspartate, tCr = total creatine, ↑ = significant increase, ↓ = significant decrease. MRS reveals significant changes in the concentration of several metabolites in APP/PS1/Ear2(-/-) mice *in vivo.*

**
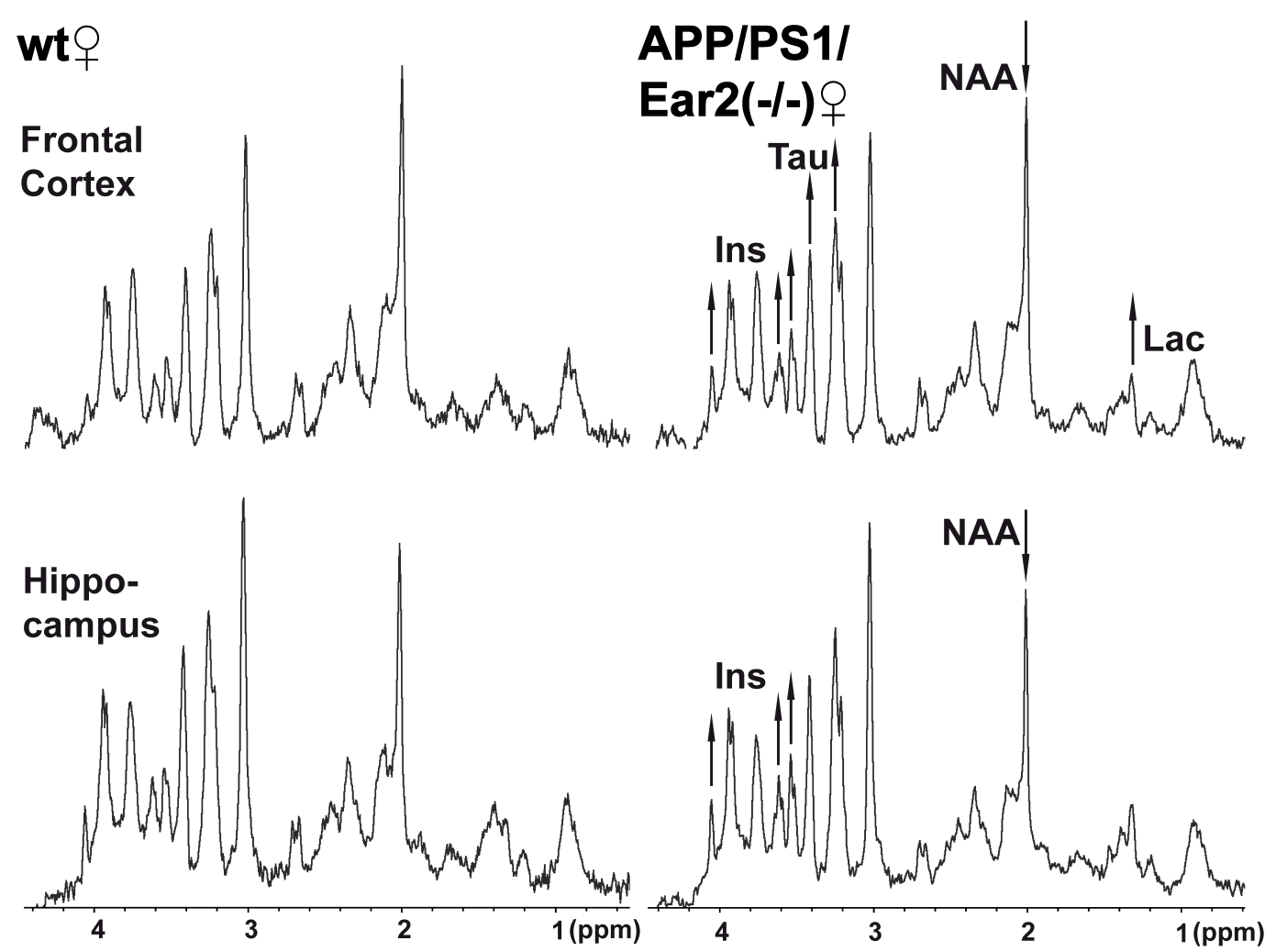
**

**Supplementary Figure 4** (Top row) MRS of the frontal cortex (STEAM, TR/TE/TM = 6000/10/10 ms, 768 averages, 1.8×1.8×1.0 mm^3^) and (bottom row) the hippocampal formation (512 averages, 1.8×1.8×1.2 mm^3^) in (left column) wild-type (n=9) and (right column) APP/PS1/Ear2(-/-) female mice *in vivo* (n=7). MRS reveals significant changes in the concentration of several metabolites in female APP/PS1/Ear2(-/-) mice *in vivo.* Exact values and ratios are summarized in Supplementary Table 2. Ins = myo-inositol, Lac = lactate, NAA = *N*-acetylaspartate, Tau = taurine, ↑ = significant increase, ↓ = significant decrease.

|  | n | Age (months) | Body Weight (g) | Brain (μl) | Brain/Intracranial Volume (%) | Ventricles (μl) | Ventricles/Intracranial Volume (%) | Intracranial Volume (μl) |
| --- | --- | --- | --- | --- | --- | --- | --- | --- |
| Wild type | 4 | 21.0 ± 2.4 | 40.0 ± 10.1 | 498 ± 6.7 | 97.4 ± 1.5 | 13.5 ± 0.9 | 2.6 ± 1.5 | 511 ± 7.2 |
| Ear2(-/-) | 4 | 22.3 ± 1.0 | 30.5 ± 3.1 | 498 ± 11 | 96.6 ± 0.7 | 17.6 ± 3.9 | 3.4 ± 0.7 | 515 ± 14 |
| APP/PS1 | 4 | 19.8 ± 1.5 | 31.0 ± 4.1 | 507 ± 6.7 | 97.2 ± 0.7 | 14.6 ± 0.9 | 2.6 ± 0.7 | 522 ± 32 |
| APP/PS1/ Ear2(-/-) | 6 | 19.7 ± 1.2 | 34.3 ± 5.0 | 496 ± 13 | 96.8 ± 0.7 | 16.3 ± 3.7 | 3.2 ± 0.7 | 513 ± 14 |

**Supplementary Table 1a** Brain Volumetry of Aged Female Mice with Different Genetic Backgrounds

Intracranial Volume = Brain + Ventricles.

**Supplementary Table 1b** Volume of Each Brain Structures (μl)

|  | Forebrain | Olfactory Bulb | Hippocampus | Brain Stem | Cerebellum | Lateral Ventricle | Third Ventricle | Fourth Ventricle | Recess of the Inferior Colliculus |
| --- | --- | --- | --- | --- | --- | --- | --- | --- | --- |
| Wild type | 316 ± 9.8 | 27 ± 0.8 | 26 ± 1.3 | 99 ± 2.3 | 63 ± 1.5 | 7.9 ± 1.0 | 3.8 ± 0.2 | 1.2 ± 0.2 | 0.6 ± 0.2 |
| Ear2(-/-) | 321± 8.5 | 28 ± 1.6 | 26 ± 1.2 | 98 ± 5.1 | 62 ± 1.0 | 12 ± 4.0 | 4.2 ± 0.4 | 1.0 ± 0.2 | 0.6 ± 0.2 |
| APP/PS1 | 328 ± 24.7 | 26 ± 1.7 | 28 ± 2.8 | 96 ± 4.5 | 67 ± 2.7 | 9.0 ± 2.9 | 3.9 ± 0.6 | 1.1 ± 0.4 | 0.6 ± 0.1 |
| APP/PS1/ Ear2(-/-) | 325 ± 8.0 | 26 ± 0.9 | 28 ± 1.8 | 94 ± 2.7 | 63 ± 5.2 | 11 ± 3.0 | 3.7 ± 0.7 | 1.2 ± 0.2 | 0.6 ± 0.2 |

“Brain” in Supplementary Table 1a = Forebrain + Olfactory Bulb + Brain Stem + Cerebellum – Lateral Ventricle, “Ventricles” = Lateral Ventricle + Third Ventricle + Fourth Ventricle + Recess of the Inferior Colliculus.

**Supplementary Table 2** Concentration (mM) and concentration ratio of major cerebral metabolites as well as T_1_ and T_2_ relaxation times of water Protons in female APP/PS1/Ear2(-/-) and wild type control mice

|  |  | **Frontal Cortex** | |  | **Hippocampus** | |
| --- | --- | --- | --- | --- | --- | --- |
| Genotype |  | Wild type | APP/PS1/Ear2(-/-) |  | Wild type | APP/PS1/Ear2(-/-) |
| *n* |  | *n* = 9 | *n* = 7 |  | *n* = 9 | *n* = 7 |
| Age (months) |  | 20.3 ± 4.2 | 19.6 ± 4.6 |  | 20.3 ± 4.2 | 19.6 ± 4.6 |
| **tCr** |  | 10.4 ± 0.8 | 10.7 ± 1.1 |  | 12.3 ± 1.1 | 12.0 ± 0.5 |
| **NAA** |  | 10.7 ± 0.4 | **9.4 ± 1.2*** |  | 9.0 ± 1.0 | **7.4 ± 1.1**** |
| **NAA + NAAG** |  | 11.8 ± 0.6 | **10.5 ± 1.1**** |  | 9.7 ± 1.2 | **8.2 ± 1.1*** |
| **Glu** |  | 12.7 ± 1.0 | 11.7 ± 2.3 |  | 10.7 ± 1.4 | 9.5 ± 1.6 |
| **Lac** |  | 0.72 ± 0.6 | **2.1 ± 1.1*** |  | 1.3 ± 0.7 | 2.0 ± 0.8 |
| **Ins** |  | 3.8 ± 0.7 | **5.6 ± 1.1**** |  | 5.2 ± 0.6 | 6.9 ± 1.5 |
| **Tau** |  | 9.6 ± 1.0 | **10.9 ± 1.2*** |  | 11.5 ± 1.2 | 11.4 ± 0.9 |
| **GABA** |  | 2.0 ± 0.2 | 1.8 ± 0.5 |  | 2.5 ± 0.6 | 2.0 ± 0.3 |
| **GPC** |  | 1.3 ± 0.3 | 1.7 ± 0.5 |  | 1.4 ± 0.6 | 1.3 ± 0.4 |
| **GPC + PCh** |  | 2.0 ± 0.3 | 2.1 ± 0.3 |  | 2.1 ± 0.4 | 2.0 ± 0.6 |
| **NAA/tCr** |  | 1.03 ± 0.07 | **0.88 ± 0.14*** |  | 0.73 ± 0.05 | **0.61 ± 0.09**** |
| **NAA+NAAG/tCr** |  | 1.14 ± 0.10 | **0.99 ± 0.13*** |  | 0.79 ± 0.06 | **0.68 ± 0.09*** |
| **Glu/tCr** |  | 1.23 ± 0.09 | 1.09 ± 0.15 |  | 0.86 ± 0.07 | 0.78 ± 0.14 |
| **Lac/tCr** |  | 0.07 ± 0.06 | **0.19 ± 0.09*** |  | 0.11 ± 0.06 | 0.20 ± 0.10 |
| **Ins/tCr** |  | 0.37 ± 0.07 | **0.53 ± 0.10**** |  | 0.42 ± 0.04 | **0.56 ± 0.11*** |
| **Tau/tCr** |  | 0.92 ± 0.04 | **1.02 ± 0.09*** |  | 0.94 ± 0.07 | 0.96 ± 0.07 |
| **GABA/tCr** |  | 0.19 ± 0.03 | 0.17 ± 0.04 |  | 0.21 ± 0.05 | 0.17 ± 0.02 |
| **GPC/tCr** |  | 0.13 ± 0.03 | 0.16 ± 0.05 |  | 0.11 ± 0.04 | 0.10 ± 0.04 |
| **GPC+PCh/tCr** |  | 0.19 ± 0.02 | 0.20 ± 0.04 |  | 0.17 ± 0.03 | 0.17 ± 0.05 |
| ***T_1_* (s)** |  | 1.58 ± 0.13 | 1.60 ± 0.11 |  | 1.52 ± 0.10 | 1.56 ± 0.06 |
| ***T_2_* (ms)** |  | 40.0 ± 2.4 | 37.8 ± 0.4 |  | 41.2 ± 2.7 | 39.3 ± 0.9 |

*, ** = p < .05, p < .01 vs. wild type in Mann-Whitney´s U-test. For abbreviations see Table 1.
